# The Impact of Spliceosome Inhibition in *SF3B1*-Mutated Uveal Melanoma

**DOI:** 10.1167/iovs.65.12.11

**Published:** 2024-10-07

**Authors:** Josephine Q. N. Nguyen, Wojtek Drabarek, Aïsha M. C. H. J. Leeflang, Tom Brands, Thierry P. P. van den Bosch, Robert M. Verdijk, Harmen J. G. van de Werken, Job van Riet, Dion Paridaens, Annelies de Klein, Erwin Brosens, Emine Kiliç, on behalf of the Rotterdam Ocular Melanoma Study Group

**Affiliations:** 1 Department of Ophthalmology, Erasmus MC Cancer Institute, Erasmus MC Medical Center Rotterdam, CA Rotterdam, The Netherlands; 2 Department of Clinical Genetics, Erasmus MC Cancer Institute, Erasmus MC Medical Center Rotterdam, CA Rotterdam, The Netherlands; 3 The Rotterdam Eye Hospital, BH Rotterdam, The Netherlands; 4 Department of Pathology, Section Ophthalmic Pathology, Erasmus MC Cancer Institute, Erasmus MC Medical Center Rotterdam, CA Rotterdam, The Netherlands; 5 Department of Pathology, Leiden University Medical Center, ZA Leiden, The Netherlands; 6 Cancer Computational Biology Center, Erasmus MC Cancer Institute, University Medical Center Rotterdam, CA Rotterdam, The Netherlands; 7 Department of Urology, Erasmus MC Cancer Institute, University Medical Center Rotterdam, CA Rotterdam, The Netherlands; 8 Department of Immunology, Erasmus MC Cancer Institute, University Medical Center Rotterdam, CA Rotterdam, The Netherlands

**Keywords:** spliceosome complex, cancer therapy, aberrant splicing, inhibitor

## Abstract

**Purpose:**

Unfortunately, treatment of patients with uveal melanoma (UM) with metastatic disease is limited. Twenty percent of patients with UM harbor a mutation in the splicing factor gene *SF3B1*, suggesting that aberrant spliceosome function plays a vital role in tumorigenesis. Splicing inhibitors exploit the preferential sensitivity of spliceosome-compromised leukemic cells to these compounds.

**Methods:**

We studied the effect of the splicing inhibitor E7107 using two UM cell lines and ex vivo cultured *SF3B1-* and *BAP1*-mutated primary UM tumor slices. These UM cell lines and ex vivo tumor slices were exposed for 24 hours to different concentrations of E7107. Tumor slices were stained with hematoxylin and eosin (H&E) and incubated with BAP1, MelanA, MIB-1, and caspase-3 antisera.

**Results:**

The E7107-exposed UM cell lines exhibited decreased cell viability and increased apoptosis, with the greatest effect on *SF3B1*-mutated UM cells. A similar effect on UM tumor slices was observed upon exposure to E7107. Additionally, RNA was isolated for differential isoform expression analysis. No significant difference in isoform usage was found genome-wide. However, specific genes were differentially expressed after E7107 treatment in the *SF3B1*-mutated samples. Moreover, E7107 had the greatest effect on intron retention.

**Conclusions:**

This study indicates/suggests that mutated *SF3B1* UM cells are more sensitive to the splicing inhibitor E7107 than wild-type *SF3B1* UM cells.

Uveal melanoma (UM) is the most common primary intraocular malignancy. UM arises from melanocytes in the uvea.^1^^,^^2^ In 25% to 34% of patients with UM, the tumors metastasize within 10 years, predominantly to the liver, leading to a poor prognosis.^3^^,^^4^ The time of onset of metastatic UM can be estimated using a prognostic model because it is related to the size and genetic profile of the primary UM.^5^ Prognostic factors include mutations in genes encoding BRCA1-associated protein 1 (*BAP1*), which has the worst prognosis; splicing factor 3b subunit 1 (*SF3B1*), which has an intermediate prognosis; and eukaryotic translation initiation factor 1A X-linked (*EIF1AX*), which has the best prognosis.^6^^–^^9^ Mutations in *SF3B1* occur in approximately 15% to 25% of UM cases^10^^–^^14^ and are associated with early- and late-onset metastasis.^15^^–^^17^ Furthermore, *SF3B1*-mutated UM generates shared neoantigens that are uniquely expressed by tumor cells^18^ and have a distinct copy number variation profile compared with other UM tumors, with more aberrations at the distal ends of chromosomes and frequent loss of chromosome 6q and gain of chromosome 6p and 8q.^19^

*SF3B1*, as part of the spliceosome complex, is involved in pre-mRNA splicing. Mutations in *SF3B1* result in aberrantly spliced transcripts, which in turn can lead to the formation of aberrant proteins, nonsense-mediated decay (NMD), and downregulation of gene expression.^13^^,^^20^ An increased sensitivity of an *SF3B1*-mutated leukemia cell line to splicing inhibitors has been observed.^21^^–^^23^ Furthermore, E7107, one of many spliceosome inhibitors, has been studied in phase I trials on human solid tumors.^24^^,^^25^ E7107 is a first-in-class spliceosome inhibitor and a semisynthetic derivative of the natural fungal product pladienolide B, which was isolated from Streptomyces platensis.^26^ E7107 targets SF3B subunit 1 and blocks splicing by targeting and binding noncovalently to the SF3B subunit of the U2 snRNP complex. This prevents tight binding of the U2 snRNP complex to the pre-mRNA branch point,^27^ which results in a decrease in the mRNA transcript levels. *SF3B1*-mutated UM cells could be more sensitive to E7107 than *SF3B1* wild-type cells due to weakened binding to the branch point.^28^

To date, limited treatments, including the recently developed protein tebentafusp, which has been shown to prolong the survival of patients with metastatic UM, are available for patients with metastatic UM.^29^ However, no successful therapy has been developed for specifically *SF3B1*-mutated UM, which is a different subgroup with greater similarity to other spliceosome mutated malignancies. By specifically targeting the mutated SF3B1 spliceosome with splicing inhibitors such as E7107, tumor progression could be delayed. Because E7107 has been studied in clinical trials, we selected E7107 as a splicing inhibitor for this pilot study. Therefore, the purpose of this study was to determine both the cytotoxicity and inhibitory effect of E7107 in vitro in UM cell lines and ex vivo in UM tumors. Due to the less stringent binding of the U2 snRNP complex to the branch point, we hypothesize that the susceptibility of *SF3B1-*mutated UM to E7107 is greater than that of *SF3B1* wild-type UM. Hence, E7107 or similar compounds could have potential as a treatment modality for *SF3B1*-mutated metastatic UM.

## Methods

### Cell Culture

Two established UM cell lines (Mel202 and 92.1) were used for this study (Mel202 kindly gifted by Dr. B. Ksander, Schepens Eye Research Institute, Harvard, Boston, MA, USA, and 92.1 kindly gifted by Dr. I. de Waard-Sieblinga, Leiden University, Leiden, The Netherlands). Mel202 and 92.1 cells were cultured in RPMI-1640 medium containing L-glutamine (Gibco, Thermo Fisher Scientific, Waltham, MA, USA) supplemented with 10% fetal calf serum (FCS; Biowest, Nuaille, France) and 2% penicillin/streptomycin (P/S; Gibco, Thermo Fisher Scientific, Waltham, MA, USA). Mel202 harbors an *SF3B1* mutation (*SF3B1*^MUT^), c.1873C>T (R625G), and 92.1 harbors an *EIF1AX* mutation (*SF3B1*^WT^), c.17G>A (G6D) (both have been verified using Sanger sequencing).^30^ Mel202 was used because it is the only usable UM cell line with an *SF3B1* mutation. Two neonatal melanocyte cell lines from healthy donors (GM21807 and GM21810) and one matching (to GM21807) healthy neonatal fibroblast line (GM21811) were used as controls. Melanocytes were cultured in Medium 254 (Gibco, Thermo Fisher Scientific, Waltham, MA, USA) supplemented with 0.5% p/s (Gibco, Thermo Fisher Scientific, Waltham, MA, USA). Fibroblasts were cultured in DMEM (1 × ) (Gibco, Thermo Fisher Scientific, Waltham, MA, USA) supplemented with 10% fetal calf serum (FCS; Biowest, Nuaille, France) and 1% penicillin/streptomycin (P/S; Gibco, Thermo Fisher Scientific, Waltham, MA, USA). The cells were grown at 37°C in a 5% CO2 humidified atmosphere (Heracell 150 CO2 incubator; Heraeus, Hanau, Germany).

### Cell Viability and Density Analysis

To determine the cytotoxic effect of E7107, the viability of the *SF3B1*^MUT^ and the *SF3B1*^WT^ UM cell lines was assessed after treatment with different concentrations of E7107 (gifted by H3 Biomedicine Inc., Cambridge, MA, USA). To evaluate the toxicity effects of DMSO as a solvent, we also used a negative control (0 nM E7107) to assess solvent toxicity. The *SF3B1*^MUT^ and the *SF3B1*^WT^ UM cell lines were seeded (2.0 × 10^5^ per well in 6-well plates) for 24 hours before exposure for 24 hours to E7107 (concentrations ranging from 0–5, 7, 10, and 20 nM). Melanocytes and fibroblasts were also seeded (9.0 × 10^4^ and 1.5 × 10^5^ per well in 6-well plates, respectively) for 24 hours before exposure to E7107. Cell viability was assessed by counting the number of viable cells per condition in triplicate with trypan blue dye (#1450021; Bio-Rad, Hercules, CA, USA) using a TC20TM Automated Cell Counter (Bio-Rad, Hercules, CA, USA). To determine the timing of the cytostatic effect of E7107, the cell density of the *SF3B1*^MUT^ and the *SF3B1*^WT^ UM cell lines was assessed during 24 hours of incubation with E7107. This effect was assessed in two different time-lapse experiments. In the first experiment, cells (5.0 × 10^3^ per well in black 96-well plates) treated with E7107 (0–20 nM) were imaged on an Opera Phenix High Content Screening System (PerkinElmer, Waltham, MA, USA) using a 10 × air objective (0.3 NA) for a period of 21 hours with a time interval of 3 hours. In the second experiment, the cells (2.0 × 10^3^ per well in black 96-well plates) were treated with E7107 (0–0.5 nM), and images were taken for a period of 69 hours with a time interval of 3 hours for the first 24 hours and then for an additional 6 hours. The captured images were analyzed using the associated Harmony office software (PerkinElmer, Waltham, MA, USA). The software was used to calculate the percentage decrease or increase in the cell area compared to that at 0 hours after each interval.

### Tumor Tissue Work-Up

Fresh UM tissue was collected from patients with UM undergoing enucleation at the Erasmus MC University MC Rotterdam and the Rotterdam Eye Hospital Rotterdam, The Netherlands, from September 2018 to September 2021. Twenty tumor tissues were collected at the Department of Pathology. Patients with iris melanoma and irradiated tumors were excluded from this study. Mutational status was determined by BAP1 immunohistochemistry, Sanger sequencing, and/or next-generation sequencing.^31^^,^^32^ The tissues were embedded in 4% agarose at a low gelation temperature (#A9414-100G; Sigma Aldrich, St. Louis, MO, USA) at 37°C before automated tissue slicing using a Leica VT 1200S Vibratome (Leica Biosystems Inc., Buffalo Grove, IL, USA) with a 0.8 mm/s slicing speed, 300 µm slice thickness, and 2.0 mm vibration amplitude. Slices were cultured in 6-well plates in culture medium (RPMI-1640 medium containing L-glutamine (Gibco, Thermo Fisher Scientific, Waltham, MA, USA) supplemented with 10% FCS (Biowest, Nuaille, France) and 2% penicillin/streptomycin (P/S; Gibco, Thermo Fisher Scientific, Waltham, MA, USA) supplemented with 0, 1, or 5 nM E7107 or solvent control at 37°C for 24 hours on a Stuart SSM1 mini orbital shaker (Cole-Parmer, Staffordshire, UK) at 60 rpm. Afterward, the slices were used for either RNA isolation or fixation for immunohistochemistry (IHC). The quantity and quality of the RNA were determined using a bioanalyzer (Agilent Genomics, Santa Clara, CA, USA). Tumors for which a full set (medium, 0, 1, or 5 nM E7107) of RNA with a high RNA quality (RIN > 8.0) was available were eligible for RNA sequencing.

### Reverse Transcription Polymerase Chain Reaction 

RNA isolation was performed using the miRNeasy Mini Kit (Qiagen, Hilden, Germany) according to the manufacturer's protocol, and cDNA was synthesized with 1 µg of RNA using the iScript cDNA Synthesis Kit (Bio-Rad Laboratories, Veenendaal, The Netherlands) according to the manufacturer's protocol. Custom primers for the exons adjacent to the splicing aberration were designed using primer 3 (Supplementary Table S1) for reverse transcription PCR (RT‒PCR; Biometra TAdvanced PCR Thermocycler) for 5 candidate genes, namely, Armadillo Repeat Containing 9 (*ARMC9*), Enolase Superfamily Member 1 (*ENOSF1*), Diphthamide Biosynthesis 5 (*DPH5*), Dihydrolipoamide S-Succinyltransferase (*DLST*), and Cyclin-dependent kinase 2 (*CDK2*). Four of these genes (*ARMC9*, *ENOSF1*, *DPH5*, and *DLST*) have been shown to be aberrantly spliced in the *SF3B1*^MUT^ UM cell lines.^13^ After reanalyzing the UM RNA-seq data of Wojtek et al.,^17^ we included one additional gene, *CDK2*, and two control genes, Charged Multivesicular Body Protein 2A (*CHMP2A*) and ER Membrane Protein Complex Subunit 7 (*EMC7*). The results were analyzed using the image analysis software ImageQuantTL to determine the effect of E7107 on splicing inhibition in aberrant transcripts and canonical transcripts. Here, transcripts were defined as “canonical” when the transcript lengths of known isoforms were unaffected. Linear regression analysis was used to determine the degree of splicing inhibition evoked by E7107 by calculating the slope.

### RNA Sequencing and Analysis

First, strand-specific cDNA libraries were generated with the strand-specific NEBNext Ultra II Directional RNA Library Prep Kit protocol and polyA mRNA workflow (NEB #E7760S/L) on an Illumina NovaSeq 6000 (Illumina, San Diego, CA, USA). Sample preparation was performed according to the manufacturer's protocol (GenomeScan, Leiden, The Netherlands). Quality control and read trimming were performed with Trim Galore version 0.6.7.^33^ Read alignment, transcript quantification, and differential expression analysis were performed using CLC Genomics Workbench version 20 (Qiagen, Hilden, Germany). Reads were aligned to the human reference genome (hg_g1k_v37 and hg38_p14) according to the following settings: mismatch cost 2, insertion/deletion cost 3, length fraction 0.8, similarity fraction 0.8, and alignment to gene regions only. Transcripts, including mitochondrial, ribosomal RNA, and microRNA, were excluded from the analysis. Paired reads were counted as one. The alignment metrics can be found in the Supplementary Material (Supplementary Table S2, Supplementary Fig. S1). The trimmed mean per million (TMM) values were used to normalize the sequencing depth across samples. For each gene, counts per million (CPM) and transcripts per million (TPM) were calculated. Total read counts can be found in the Supplementary Material (see Supplementary Table S2).

Differential isoform expression analysis was performed using the Galaxy genome-wide alternative splicing analysis tool.^34^^–^^36^ In addition, Find RAre Splicing Events in RNA_seq (FRASER) was used to determine potential candidates for aberrant splicing events at the cohort level.^37^ Here, the same ΔΨ cutoff was applied for expression filtering. For the computation of the *P* and z values, ψ5 (alternative acceptors) = 3, ψ3 (alternative donors) = 5, and θ (splicing efficiencies) = 2 were used. The results for individual samples were ranked according to their ΔΨ values, and the top 25 genes of each sample were combined for comparison based on their psi/theta values. Afterward, candidates were included when a decrease in the ΔΨ value was assessed per set, because this showed that a decrease in aberrant transcript formation occurs when treated with E7107.

### Immunohistochemistry 

After formalin fixation, the tumor slices were embedded in paraffin, and 4 µm sections were generated for microscopic analysis using a microtome. The IHC was performed with an automated, validated, and accredited staining system (Ventana Benchmark ULTRA, Ventana Medical Systems, Tucson, AZ, USA) using an Ultraview or Optiview Universal Alkaline Phosphatase Red Detection Kit (#760-501). Following deparaffinization and heat-induced antigen retrieval, the tissue samples were incubated according to their optimized incubation time with the antibody of interest (Supplementary Table S3). The cells were incubated with hematoxylin II counterstain for 12 minutes and then with blue coloring reagent for 8 minutes to turn the purple hematoxylin blue, according to the manufacturer's instructions (Ventana Benchmark ULTRA, Ventana Medical Systems, Tucson, AZ, USA). We used hematoxylin and eosin (H&E) to examine the histological tumor architecture. The IHC for melanoma antigen (MelanA) was performed to identify areas with > 90% tumor cells, and BAP1^31^ immunostaining was used to determine whether tumors were BAP1 positive or negative, thereby indicating whether a *BAP1* mutation was present. Immunohistochemical detection of the proliferation marker Ki67 was performed using the monoclonal antibody mind bomb E3 ubiquitin protein ligase 1 (MIB-1) to evaluate areas containing > 90% tumor cells. Similarly, caspase-3 immunostaining was used to detect apoptotic cells.

### Statistical Analysis

Statistical analysis was performed using GraphPad Prism 9.0 (GraphPad Software, Inc., San Diego, CA, USA). Two-way ANOVA with Tukey's test or paired Student's *t*-test was used to compare differences between groups and concentrations. Linear regression was performed to determine the degree of splicing inhibition. The lethal concentration (LC) 50 value was defined as the concentration at which the cell viability decreased by 50%. The mean number of positive cells detected by IHC on the slices was calculated per mm^2^. When slices were smaller than 1 mm^2^, the mean number of positive cells was scaled to the correct size for the analysis. A *P* value of < 0.05 was considered to indicate statistical significance.

## Results

### Cytotoxicity and Proliferation Inhibition of E7107 In Vitro

**Figure 1.**
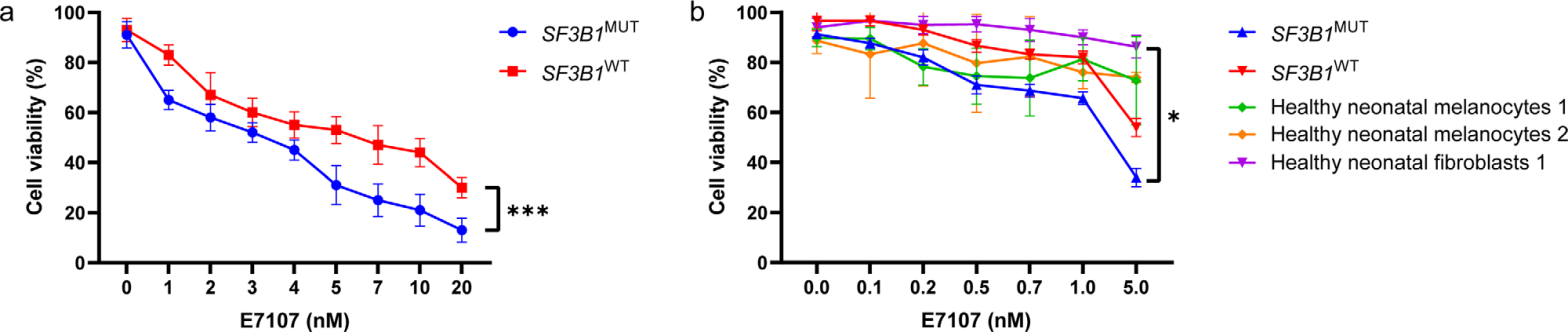
**The effects of E7107 on cell viability in uveal melanoma and control cell lines.** (**a**) Mel202 *SF3B1*^MUT^ and 92.1 *SF3B1*^WT^ UM cell lines were treated with 0-20 nM E7107 for 24 hours to assess the difference between wild-type and mutated *SF3B1*. (**b**) Mel202 *SF3B1*^MUT^ and 92.1 *SF3B1*^WT^ UM cell lines and healthy melanocytes and fibroblasts were treated with 0 to 5 nM E7107 for 24 hours to assess the difference between uveal melanoma cell lines and healthy normal cell lines. Paired Student's *t*-test and 2-way ANOVA were used to calculate the statistical significance between both cell lines and between concentrations, respectively. A statistically significant difference between both cell lines was observed at all concentrations. Compared to medium and 0 nM E7107, 2 nM E7107 had a statistically significant effect on both cell lines. * = *P* < 0.05; *** = *P* < 0.001; (*N* > 3).

**Figure 2.**
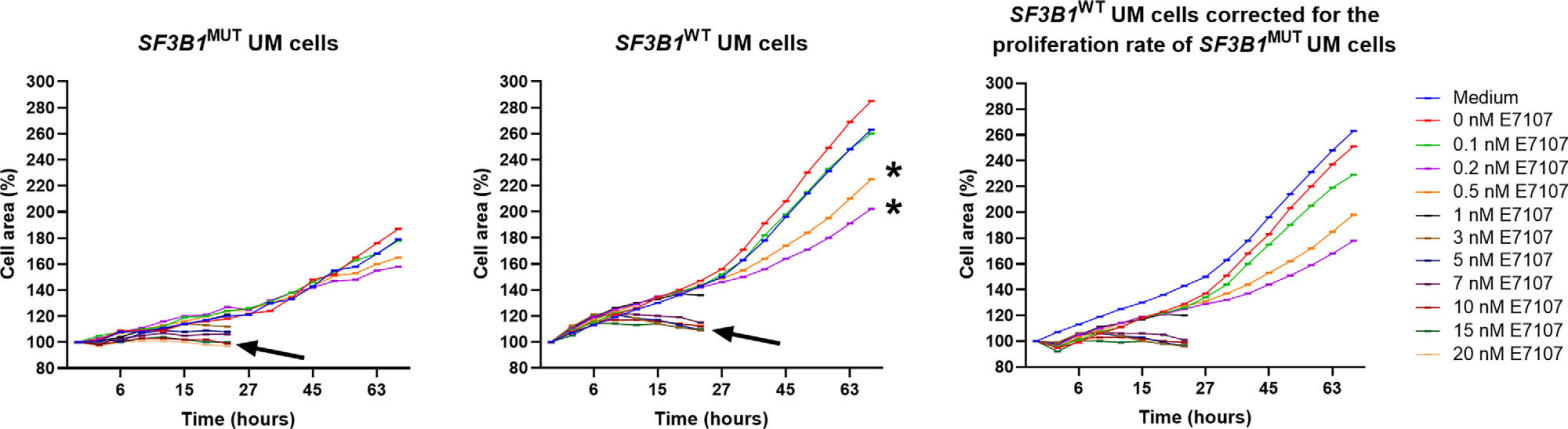
**Proliferation rate inhibition after exposure to E7107.** Drug potency was determined in *SF3B1*^MUT^ and *SF3B1*^WT^ UM cell lines using the Opera Phenix High Content Screening System. A significant decrease in proliferation inhibition was observed after 21 hours of treatment with 10 nM E7107 (*arrow*) or more (*P* = 0.0007) in the Mel202 *SF3B1*^MUT^ cells and 15 nM E7107 (*arrow*) in the 92.1 *SF3B1*^WT^ cell line. After 69 hours, no significant differences were observed in the Mel292 *SF3B1*^MUT^ cell line, whereas 0.2 and 0.5 nM E7107 (*asterisk*) had significant effects on the 92.1 *SF3B1*^WT^ cell line (*P* = 0.0019). The proliferation rate of the 92.1 *SF3B1*^WT^ cell line was subsequently corrected for the reported doubling rate of the Mel202 *SF3B1*^MUT^ cell line.^30^

The cytotoxicity of a single dose of E7107 was assessed in the Mel202 *SF3B1*^MUT^ and 92.1 *SF3B1*^WT^ cell lines. A decrease in cell viability was observed when E7107 was administered in a dose-dependent manner to both cell lines (Fig. 1a). The decrease in cell viability was statistically significant after treatment with 2 nM E7107 compared to that after treatment with medium or 0 nM E7107 in both cell lines (*P* < 0.05). Furthermore, the toxicity was significantly greater in the Mel202 *SF3B1*^MUT^ cell line (*P* = 0.0004). The LC50 values were 2.66 nM and 5.67 nM in the Mel202 *SF3B1*^MUT^ and the 92.1 *SF3B1*^WT^ UM cell lines, respectively, and the cytotoxic potency of E7107 was 2.1 times greater in the Mel202 *SF3B1*^MUT^ cell line than in the 92.1 *SF3B1*^WT^ cell line. Next, the difference in the cytotoxicity of E7107 between UM cell lines and control cell lines was assessed (Fig. 1b). A decrease in cell viability was observed in a smaller concentration range in all cell lines, mostly in the Mel202 *SF3B1*^MUT^ UM cell line, compared to healthy neonatal fibroblasts (*P* = 0.0142). Proliferation was inhibited in both the Mel202 *SF3B1*^MUT^ and the 92.1 *SF3B1*^WT^ UM cell lines after treatment with E7107 in a time- and dose-dependent manner (Fig. 2). In both cell lines, DMSO (0 nM E7107) seemed to have a positive effect on proliferation. The cells were still proliferative until they were treated with 0.5 to 1 nM E7107, at which point the proliferation stopped. A significant decrease in cell proliferation was detected after 21 hours in the Mel202 *SF3B1*^MUT^ cell line treated with 10 nM E7107 or more (*P* = 0.0007), whereas only 15 nM E7107 had a significant effect on proliferation in the 92.1 *SF3B1*^WT^ cell line (*P* = 0.0089). After 69 hours, no significant differences were observed in the Mel202 *SF3B1*^MUT^ cell line, whereas the 92.1 *SF3B1*^WT^ cell line was significantly inhibited with concentrations of 0.2 and 0.5 nM E7107 (*P* = 0.0019). Due to different doubling time between both the Mel202 *SF3B1*^MUT^ and the 92.1 *SF3B1*^WT^ cell lines,^30^ we have corrected the proliferation rate for the doubling time (see Fig. 2).

### Transcription Level Analysis

**Figure 3.**
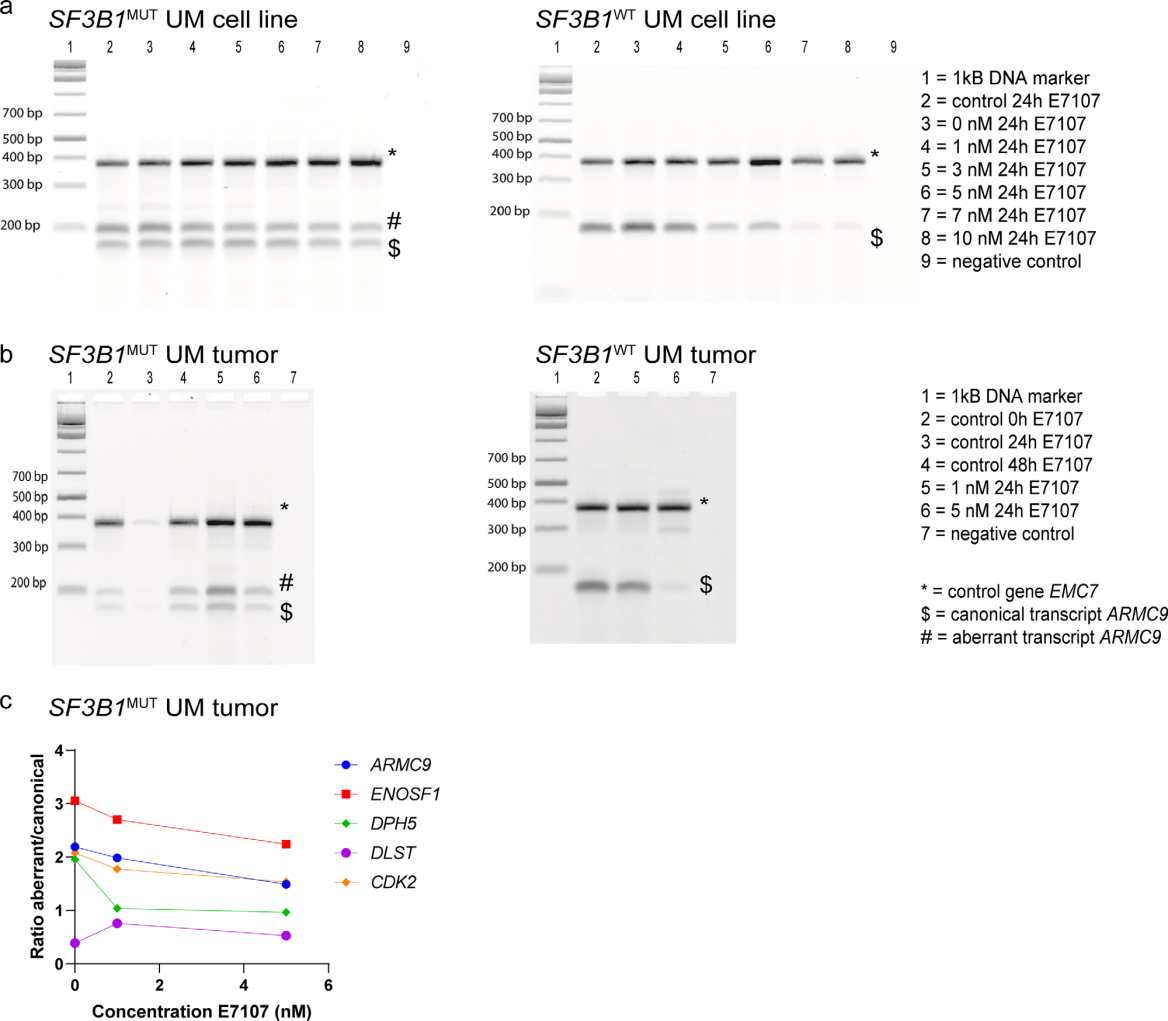
**RT‒PCR analysis of the effect of E7107 treatment on the splicing inhibition of UM cell lines and tumors.** Multiplex RT‒PCR products for *ARMC9* in the Mel202 *SF3B1*^MUT^ and the 92.1 *SF3B1*^WT^ cell lines (**a**) and tumors (**b**) treated with different concentrations of E7107. Amplification of the *SF3B1*^MUT^-sensitive genes resulted in both aberrant and canonical transcript formation for both *SF3B1*^MUT^ cell line and tumors, whereas the *SF3B1*^WT^ products only displayed normal transcript formation. (**c**) Aberrant and canonical transcript formation in *SF3B1*^MUT^ tumors was quantified using ImageQuantTL. The ratio of aberrant versus canonical transcripts for various genes in *SF3B1*^MUT^ tumor tissue. The insensitive gene *EMC7* was used as a control for both cDNA quality and quantity. *SF3B1*^WT^ tumors had an *EIF1AX* mutation. The analysis of other genes can be found in the Supplementary Material (Supplementary Fig. S3).

The sensitivity of E7107 to splicing inhibition was assessed for 5 target genes of interest (*ARMC9*, *ENOSF1*, *DPH5*, *DLST*, and *CDK2*) and 2 control genes (*CHMP2A* and *EMC7*) in both the Mel202 *SF3B1*^MUT^ and the 92.1 *SF3B1*^WT^ cell lines. Due to the mutation in *SF3B1*, aberrant transcripts are present in the Mel202 *SF3B1*^MUT^ cell line. In general, a reduced transcript level is seen in both the Mel202 *SF3B1*^MUT^ and the 92.1 *SF3B1*^WT^ cell lines after E7107 treatment (Fig. 3a). Both the canonical and the aberrant transcripts were affected, yet a greater decrease in the amount of aberrant, compared to canonical, transcripts from the genes of interest was observed within the Mel202 *SF3B1*^MUT^ cell line (Supplementary Fig. S3).

### Tumor Tissue

Fresh UM tissues from 20 patients undergoing enucleation were collected, and the mutational status of key UM-related hotspot genes for all tumors was determined (Supplementary Table S4). Seventeen tumors harbored a primary driver mutation in G protein Subunit Alpha Q (*GNAQ*) or G protein Subunit Alpha 11 (*GNA11*), in one tumor, a mutation in Cysteinyl Leukotriene Receptor 2 (*CYSLTR2*) was detected; and, in one other tumor, a mutation in Phospholipase C Beta 4 (*PLCB4*) was detected. In almost all tumors, a secondary driver mutation was detected: 10 patients harbored a mutation in *BAP1* and/or were BAP IHC negative, 3 in *SF3B1* and 5 in *EIF1AX*.

### Ex Vivo Splicing Inhibition Assay

The inhibitory effect of E7107 on the splicing of UM tissue slices was assessed via RT‒PCR using the same genes used in our in vitro experiments. The formation of both canonical and aberrant transcripts was observed in *SF3B1*^MUT^ tumors (tumor 01), whereas only canonical transcripts formed in *SF3B1*^WT^ tumors (tumor 03; Fig. 3b). RT-PCR analysis showed no evident decrease in ratio of aberrant versus canonical transcripts formation for most genes, although the ratio was lowest at a concentration of 5 nM E7107 (Fig. 3c).

### RNA Sequencing and Splicing Analysis

**Figure 4.**
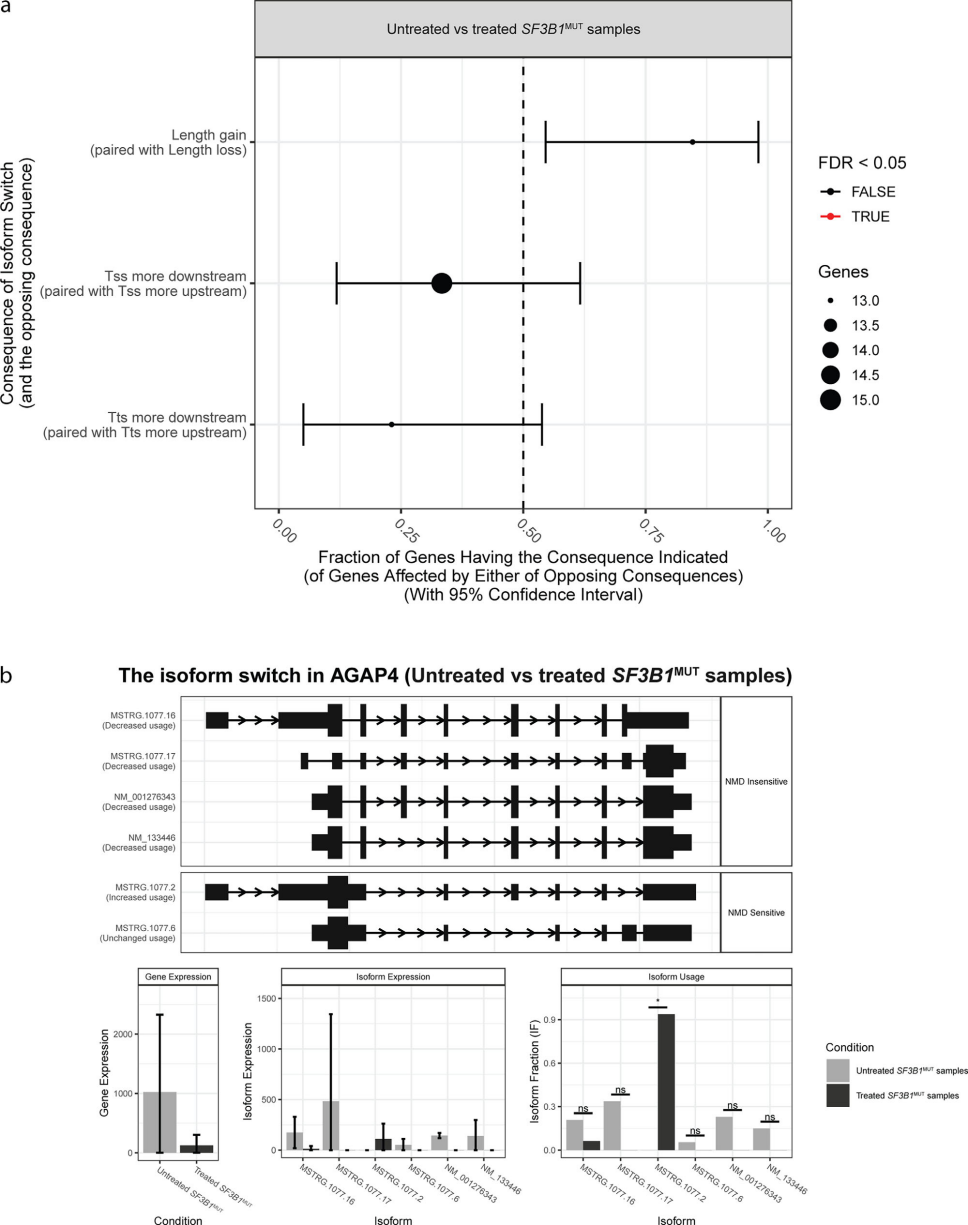
**(a) Enrichment and depletion of isoform switches.** The x-axis shows the fraction (with 95% confidence interval) resulting in the consequence indicated on the y-axis in the switches from untreated to treated *SF3B1*^MUT^ tumor samples. *SF3B1*^MUT^ tumor samples treated with E7107 were enriched in isoform length gain but not downstream of the transcription start site or transcription termination site. The *dashed line* indicates no enrichment or depletion. The color indicates an FDR < 0.05 (*red*) or > 0.05 (*black*). FDR, false discovery rate; Tss, transcription start site; Tts, transcription termination site. (**b**) Isoform expression profile plot of *AGAP4*. The plot integrates isoform structures along with the annotation, gene and isoform expression, and isoform usage. Decreased *AGAP4* gene expression was observed after E7107 treatment, as was significant differential expression of the isoform MSTRG.1077.6.

Next, RNA sequencing was performed on untreated and treated E7107 *SF3B1*^MUT^ and *SF3B1*^WT^ tumor samples. The patterns of alternative splicing and gene-specific isoform switching (IS) in the samples were assessed. No differences were found in alternative splicing in the *SF3B1*^WT^ tumor samples. In the *SF3B1*^MUT^ tumor samples, genome-wide IS events resulting in gain or loss of a specific consequence were analyzed. Although some types of consequences seemed to be enriched or depleted after E7107 treatment, this difference was not statistically significant (Fig. 4a). Furthermore, no significant genome-wide difference in isoform usage was observed after the *SF3B1*^MUT^ tumor samples were treated with E7107 (Supplementary Fig. S4). One example of a gene with isoform switching with predicted functional consequences is *AGAP4* (Fig. 4b; Supplementary Table S5). *AGAP4* expression decreased after E7107 treatment. Treatment with E7107 resulted in the expression of a specific new *AGAP4* isoform, MSTRG.1077.2.

**Figure 5.**
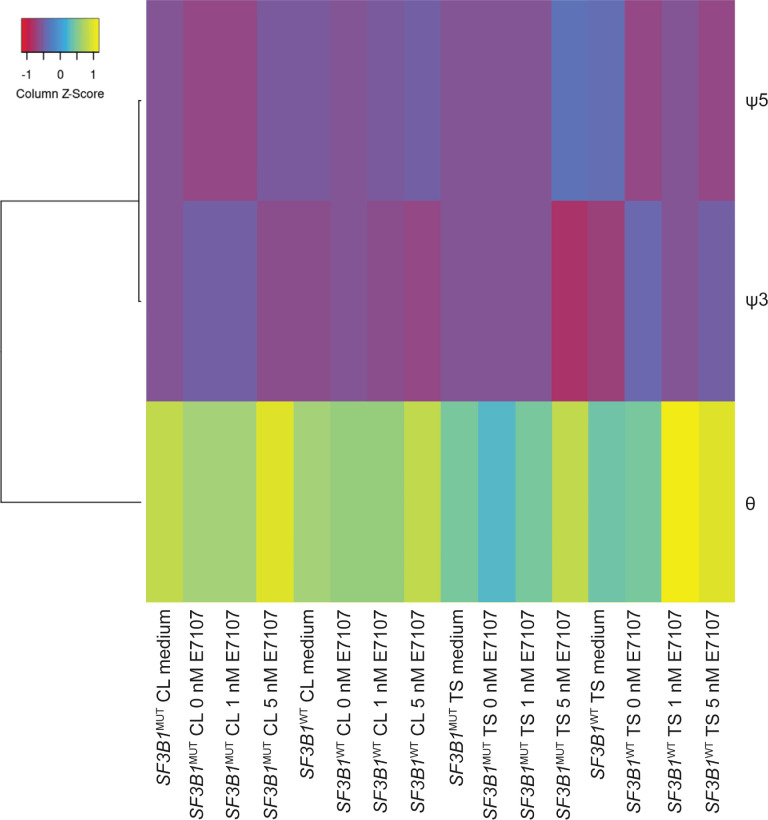
**Aberrant splicing analysis.** Heatmap of aberrantly spliced genes per sample per event: intron retention (θ), aberrant donor site usage (ψ3), and aberrant acceptor site usage (ψ5). A greater number of intron retentions was observed than of aberrant donor/acceptor site usage events. The intron retentions were most often observed in 5 nM E7107 samples. CL, cell line; TS, tissue slice.

Next, aberrant splicing events were assessed with FRASER analysis at the cohort level. Aberrant splicing events were categorized into three event groups: intron retention (θ), aberrant donor site usage (ψ3), and aberrant acceptor site usage (ψ5). After UM, the Mel202 *SF3B1*^MUT^ and the 92.1 *SF3B1*^WT^ cell lines as well as the *SF3B1*^MUT^ and *SF3B1*^WT^ tumors were treated with E7107, aberrant splicing with intron retention events were most often assessed (mostly in 5 nM E7107 samples), followed by aberrant acceptor site usage and aberrant donor site usage (Fig. 5). To assess the effects on proliferation and viability at the protein level, we next detected positive cells in tumor slices via IHC.

### IHC Staining

**Figure 6.**
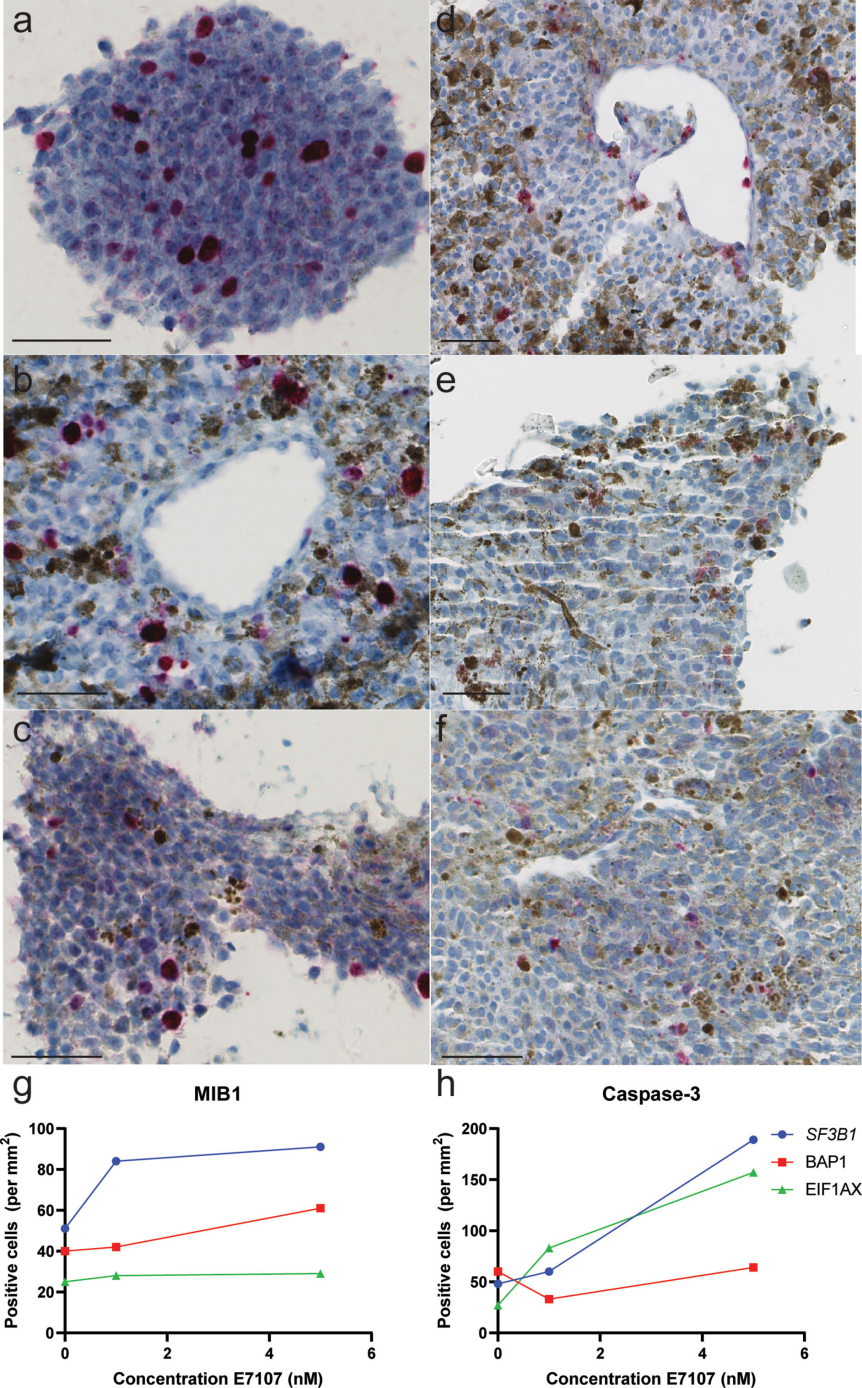
**Immunohistochemical staining of UM tumor tissues treated with 5 nM E7107.** Proliferating cells were marked with mind bomb E3 ubiquitin protein ligase 1 (MIB-1) in (**a**) *SF3B1*^MUT^ tumors (tumor 01), (**b**) *BAP1*^MUT^ tumors (tumor 19), and (**c**) *EIF1AX*^MUT^ tumors (tumor 03). An increase in the number of apoptotic cells was detected in (**d**) *SF3B1*^MUT^ tumors (tumor 14), (**e**) *BAP1*^MUT^ tumors (tumor 02), and (**f**) *EIF1AX*^MUT^ tumors (tumor 03). with an anti-caspase-3 antibody (scale bar 50 µm). The mean number of MIB-1- and caspase-3-positive cells per mm^2^ is shown (**g**, **h**).

The morphology and detection of tumor cells were assessed with H&E and Melan-A staining, respectively. More apoptotic cells were observed after E7107 treatment. There was a general trend toward an increase in apoptotic cells (caspase-3) in all tumor slices, with the greatest increase in the *SF3B1*^MUT^ tumor slices treated with 5 nM E7107 (Fig. 6). A slight increase in proliferation (MIB-1) was detected in all the samples (*P* = 0.4958).

## Discussion

In this study, we assessed the in vitro cytotoxicity and splicing inhibitory effects of E7107 on UM cell lines and its ex vivo effects on UM tumor tissue slices. Although splicing is an essential process in all mammalian cells, this study suggested that the viability of *SF3B1*^MUT^ UM is reduced in response to the splicing inhibitor E7107 compared to that of *SF3B1*^WT^ UM.

Our results suggest that the splicing inhibitor E7107 decreases the splicing of mRNA transcripts potentially more in the Mel202 *SF3B1*^MUT^ cell line than in the 92.1 *SF3B1*^WT^ cell line in a dose-dependent manner at an optimal concentration of 5 nM E7107, as determined by RT‒PCR. However, although quantitative PCR (qPCR) is a more precise assay than RT‒PCR, the quantification of canonical and aberrant transcripts with qPCR imposes other problems. With qPCR, two different sets of primers are needed: one set for the canonical transcript and another set for the aberrant transcript, where the primers were designed on the intron borders due to aberrant splicing. With this method, aberrant transcripts can be missed and / or can be overexpressed during analysis (Supplementary Fig. S5). However, with RT‒PCR, only one set of primers were used for both canonical and aberrant transcripts. Furthermore, due to aberrant splicing, the aberrant transcript formed may impose significant challenges to the gene stability in qPCR. These transcripts are often unstable, which can lead to inconsistent amplification, increased (reference) gene expression variability (by formation of non-functional splice variants), or different splicing patterns between samples. Therefore, because gene stability is crucial in qPCR, we have analyzed transcript formation after E7107 treatment with RT-PCR. Furthermore, in our in vitro proliferation analysis, no significant differences were observed after 69 hours in the Mel202 *SF3B1*^MUT^ cell line, whereas the 92.1 *SF3B1*^WT^ cell line was significantly inhibited by 0.2 and 0.5 nM E7107. An explanation for these skewed results could be that E7107 is more toxic in the Mel202 *SF3B1*^MUT^ cell line, where apoptosis occurs earlier in the affected cells; therefore, the results were mainly for the unaffected cells. In contrast, the 92.1 *SF3B1*^WT^ cell line could be less affected by E7107, as these cells need more exposure time to reach the same effect. Furthermore, the two cell lines used in this study (Mel202 and 92.1) have different proliferation rates and doubling times, making it difficult to interpret and compare the results.^30^ These cancer cell lines have several limitations, such as a lack of tumor heterogeneity and altered cell characteristics compared to those of the primary tumor. Using other relevant cell models, such as iPS cells, animal models and new patient-derived xenografts (PDXs), will validate and strengthen these results. Another approach would be with the use of isogenic *SF3B1* UM cell lines. Isogenic *SF3B1* UM cell lines have the advantage of having similar chromosomal characteristics and copy number variations among others, making comparison of the results easier compared to two different cell lines. With this, additional analysis such as impact on cell cycle and pathway analysis would be more accurate to interpret. Moreover, with differential isoform expression analysis in *SF3B1*^MUT^ tumor samples, significant isoform patterns with predicted functional consequences were found for several genes. However, at the genome-wide level, no significant differences in isoform usage were detected after E7107 treatment. Furthermore, no differences in isoform expression were found in *SF3B1*^WT^ tumor samples after E7107 treatment. This indicates that E7107 targets genes with specific isoforms, which hopefully has the potential to reduce cell toxicity. It was also shown that E7107 has the greatest effect on intron retention in aberrantly spliced genes, which is consistent with the literature.^22^ Because there were no biological replicates in the RNA-seq analysis due to limited material, including more samples and/or including single-cell RNA-seq data in the future would strengthen the analysis. Furthermore, we confirmed our findings on viability at the protein level via IHC. An increase in apoptotic cells was observed with E7107 treatment in all the samples. Apoptosis at lower concentrations could also be a result of the toxicity of the solvent DMSO, as DMSO can be toxic to cells under certain conditions (such as exposure for prolonged periods or when high concentrations are used).^38^ A slight increase in proliferating cells was observed in all tumors after E7107 exposure. In general, approximately 2% of UM cells are MIB-1 positive; hence, this increase in positive cells could also be due to nonspecific staining of lymphocytes.^39^ Even when > 90% of the analyzed areas had tumor cells, infiltrating lymphocytes in the tumor could not always be excluded.

The inherent sensitivity of mutated cells to E7107 and other splicing inhibitors has also been assessed in other studies.^40^^-^^44^ Obeng et al. assessed increased sensitivity to E7107 in *SF3B1*^MUT^ (K700E) murine myelodysplastic syndrome (MDS) cells in vitro and in vivo.^23^ Similar to our results, Obeng et al. showed that *SF3B1*^MUT^ murine MDS cells are more sensitive to E7107 (cell viability = 0.619 nM) than are *SF3B1*^WT^ cells (cell viability = 1.249 nM), strengthening our findings. Notably, these low values show a strikingly smaller toxicity range compared to our values of 2.66 nM in the Mel202 *SF3B1*^MUT^ and 5.67 nM in the 92.1 *SF3B1*^WT^ cell lines. Mice were intravenously injected with 4 mg/kg E7107 daily via the tail vein for 10 days (5 days on, 2 days off, and then repeated). The in vivo results indicated that the sensitivity of the mutated cells to E7107 was similar to that of the wild-type cells. Furthermore, the effects of E7107, another splicing gene, on *SRSF2* have also been evaluated.^22^ Treatment of murine myeloid leukemia cells with 4 mg/kg E7107 intravenously for 10 consecutive days preferentially resulted in the death of *SRSF2*-mutated cells, similar to the findings of the study mentioned above. Although mutations in *SRSF2* have been detected in UM, they occur less frequently than in leukemia.^45^^,^^46^ Nonetheless, E7107 only exerts its splicing inhibitory effects by binding directly to the SF3b complex. A previously identified mutation in *SF3B1* (R1074H) has been shown to be less sensitive to E7107 due to its inability to bind to the SF3b complex.^47^ Lee et al. confirmed these findings with a 300-fold greater IC_50_ in *SF3B1*^R1074H^ cells than in *SF3B1*^WT^ cells.^22^ Although mutations in *SF3B1* at R1074H have not been previously reported in UM,^48^ this underlines the importance of the specificity of E7107 in spliceosome mutant malignancies.

Here, our analysis showed a clear inhibition of cell proliferation until 21 hours in both cell lines. After that period, the effects of E7107 seemed less effective regardless of the mutation status. However, 21 hours is a short period of time, and this might be difficult for future studies to translate into preclinical trials. It would benefit to assess if the time period could be extended with similar results, possibly with different concentration ranges. Furthermore, the LC50 values in our study were greater than those in other studies.^22^^,^^23^ The LC50 values were 2.66 nM and 5.67 nM in the Mel202 *SF3B1*^MUT^ and the 92.1 *SF3B1*^WT^ UM cell lines, respectively, and the cytotoxic potency of E7107 was 2.1 times greater in the Mel202 *SF3B1*^MUT^ cell line than in the 92.1 *SF3B1*^WT^ cell line. However, it is debatable whether the difference in LC50 values observed between the *SF3B1*^MUT^ and the *SF3B1*^WT^ UM cell lines has (pre)clinical significance. It would be interesting to assess this difference in other models in addition to in vitro models. For example, PDX models of primary UM have been developed and tested with multiple drugs to select the best personalized therapy for patients with UM.^49^ The values were greater for both the *SF3B1*^MUT^ and the *SF3B1*^WT^ cell lines. This could be due to differences in cell type (human versus murine) or E7107 treatment (24 hours versus 72 hours). Another explanation could be that uveal melanoma cells are less sensitive to E7107 in vitro than other malignant cells, such as MDS cells. The in vitro concentration range of E7107 in our study was within the range of that used in other studies.^22^^,^^23^^,^^50^ Furthermore, a plateau phase was observed between 0.5 and 1 nM E7107 in our proliferation assay, which coincides with the LC50 values (0.619 – 0.90 nM) of these studies.^22^^,^^23^ Although the LC50 values of these studies varied among cell lines, they used the same dose for in vivo (4 mg/kg/day) experiments. With this dose, no evident toxicity of E7107 was mentioned in these studies. However, in contrast to UM, which has a doubling time of 154 to 511 days (63 days in metastatic UM), murine leukemic cells have a doubling time of only approximately 0.5 days.^51^^–^^53^

To date, E7107 has been studied in 2 separate phase I clinical trials in patients with solid tumors. In both trials, E7107 reversibly inhibited pre-mRNA in a dose-dependent manner at a maximum tolerated dose of 4.0 to 4.3 mg/m^2^.^24^^,^^25^ E7107 was administered intravenously, with a bolus of 5 to 30 minutes on days 1, 8, and 15^24^ and on days 1 and 8.^25^ In both studies, E7107 had a high systemic clearance, with a comparable plasma elimination half-life (Eskens et al.: 5.3–15.1 hours and Hong et al.: 6–13 hours). The mRNA levels of selected target genes, monitored via peripheral blood, showed a reversible 15- to 25-fold decrease as well as increased levels of unspliced pre-mRNAs of target genes. One patient had a decreased size and number of hepatic metastases after treatment, whereas another patient with pancreatic cancer had a 10% decrease in the sum of the largest tumor diameter and a reduction in cancer antigen 19-9 levels. Furthermore, after the discontinuation of E7107 administration, stable disease was observed, with no changes in the size of the metastases after more than 3 months of follow-up in up to 31% of the patients. E7107 was generally well tolerated, with the most common drug-related adverse events being gastrointestinal-related events, such as nausea and vomiting. However, visual impairment was reported in 3 out of 66 patients, which resulted in the termination of both trials. All patients experienced bilateral central scotomas, and after the discontinuation of E7107, the first patient had no progression and experienced partial recovery. However, the second patient experienced vision deterioration to nearly complete blindness, with extensive pallor and cupping. The full-field electroretinogram was normal, and vision loss was most likely caused by optic nerve dysfunction. The third patient most likely had bilateral optic neuritis based on extensive ophthalmologic investigations, including HFA 24-2 visual field analysis and Goldmann perimetry, Electroretinography, and visual evoked potential measurements. After high-dose corticosteroid treatment, the symptoms improved, with only mild residual symptoms. Because our study evaluated the inhibitory effects in vitro and ex vivo, this toxicity could not be assessed. It remains unclear whether the observed toxicity in both trials is due to SF3B1 inhibitors in general or specifically to E7107. Nonetheless, this toxicity should be assessed extensively before applying E7107 to patients with UM because these patients either have lost one eye or may have pre-existent visual impairment due to radiation therapy. To optimize its efficacy, compounds similar to E7107 could be used with an altered administration method, for example, nanoparticles for direct delivery to the liver for greater efficacy,^54^ or in combination with other strategic compounds. Due to aberrant splicing in *SF3B1*-mutated UM, shared neoantigens that are uniquely expressed by tumor cells have been generated.^18^ This can lead to the recognition and killing of these cells by specific CD8+ T cells, which can be used as therapeutic strategies, possibly independent of the *SF3B1* mutation status.^55^

Recently, a phase I clinical trial in myeloid neoplasia, including MDS, was successfully conducted on the splicing inhibitor H3B-8800 (chemically similar to E7107).^56^ Similar to E7107, H3B-8800 has also shown preferential sensitivity toward spliceosome mutant cells.^57^^,^^58^ The study demonstrated that this drug has acceptable adverse effects, and no visual impairment was observed during the study.^56^ Interestingly, they identified a subset of patients who could benefit from this treatment. These patients all had an *SF3B1* mutation. Furthermore, synthetic introns targeting specific alternative transcripts that are spliced only in *SF3B1*^MUT^ cells but are otherwise unspliced in *SF3B1*^WT^ cells have also been demonstrated to complement other synthetic therapeutic options.^59^ In CLL cell lines, the combination of H3B-8800 with the BCL2 inhibitor venetoclax had a synergistic effect on cell lethality,^58^ similar to the effect of the combination of E7107 and venetoclax.^60^ Venetoclax has previously been assessed in UM as well,^61^ making this an interesting therapeutic combination option. Unfortunately, we were unable to obtain H3B-8800 from the manufacturer as the company no longer exists, and therefore assessing H3B-8800 in (*SF3B1*^MUT^) UM is not possible at present. However, assessing future available splicing inhibitors with and without other compounds will strengthen our results. Another interesting combination can include PARP inhibitors. Mutations in *SF3B1* (K700E mutation) have been shown to induce a BRCA-like cellular phenotype in leukemic cells, which is synthetically lethal to PARP inhibitors.^62^ PARP inhibitors have been used in several malignancies and have been studied in UM previously.^63^ Moreover, *SF3B1*^MUT^ cells are sensitive to PARP inhibitors, independent of the hotspot mutation and tumor site^64^; however, no changes in alternative splice site 3′ recognition were detected, and minor transcriptional changes were detected after exposure to PARP inhibitors. This finding suggested that PARP inhibitors do not alter global splicing in cells. In vivo, reduced tumor growth and metastatic formation were observed in mice bearing Mel202 *SF3B1* mutant (R625H) cells.^64^ Additionally, up to one-third of aberrant transcripts in the Mel202 *SF3B1*^MUT^ cells are more translated than canonical transcripts, and most of these genes are enriched in metabolic functions.^65^ Considering that translated aberrant transcripts presumably contribute to the disruption of cellular processes, assessing these genes and metabolic pathways may be interesting for exploring new targets in *SF3B1*^MUT^ UM. *SF3B1*^MUT^ cells exhibit decreased mitochondrial respiration and promoted glycolysis, suggesting that these cells trigger a metabolic switch toward glycolysis to compensate for defective mitochondrial metabolism (often observed in tumors).^66^^,^^67^ *SF3B1*^MUT^ cells therefore also display increased sensitivity to glycolysis inhibitors compared to *SF3B1*^WT^ cells. In addition, the use of synthetic introns has also been explored in *SF3B1*^MUT^ cancer cells, including UM cells.^59^ These synthetic introns are efficiently spliced in *SF3B1*^MUT^ cells but not in *SF3B1*^WT^ cells, resulting in the formation of mutation-specific proteins. They can thus be explored for their ability to complement other (protein)-targeted compounds. These findings support our suggestion to combine availably splicing inhibitors with other compounds for synergistic effects in *SF3B1*^MUT^ UM. Overall, we explored the therapeutic potential of targeted compounds, similar to E7107, for specific splicing point mutations that may have clinical relevance for patients with UM with splicing mutations.

## Conclusions

In this pilot study, we have assessed splicing inhibitor E7107 in UM. We have demonstrated a decrease in cell viability and aberrant transcript formation of target genes in *SF3B1*^MUT^ UM compared to *SF3B1*^WT^ UM. This shows that splicing inhibitors might be a therapeutic option in spliceosome mutant malignancies, including *SF3B1*^MUT^ UM. Further research into the in vivo efficacy and toxicity of splicing inhibitors, as well as combination with other compounds is warranted.

## Supplementary Material

Supplement 1

## References

[1] Virgili G, Gatta G, Ciccolallo L, et al. Incidence of uveal melanoma in Europe. *Ophthalmology*. 2007; 114: 2309–2315. 17498805 10.1016/j.ophtha.2007.01.032

[2] Jager MJ, Shields CL, Cebulla CM, et al. Uveal melanoma. *Nat Rev Dis Primers*. 2020; 6: 24. 32273508 10.1038/s41572-020-0158-0

[3] Shields CL, Furuta M, Thangappan A, et al. Metastasis of uveal melanoma millimeter-by-millimeter in 8033 consecutive eyes. *Arch Ophthalmol*. 2009; 127: 989–998. 19667335 10.1001/archophthalmol.2009.208

[4] Diener-West M, Reynolds SM, Agugliaro DJ, et al. Development of metastatic disease after enrollment in the COMS trials for treatment of choroidal melanoma: collaborative ocular melanoma study group report no. 26. *Arch Ophthalmol*. 2005; 123: 1639–1643. 16344433 10.1001/archopht.123.12.1639

[5] Rola AC, Kalirai H, Taktak AFG, et al. A retrospective analysis of 10 years of liver surveillance undertaken in uveal melanoma patients treated at the supraregional “Liverpool Ocular Oncology Centre”, UK. *Cancers (Basel)*. 2022; 14: 2187. 35565316 10.3390/cancers14092187 PMC9102800

[6] Yavuzyigitoglu S, Koopmans AE, Verdijk RM, et al. Uveal melanomas with SF3B1 mutations: a distinct subclass associated with late-onset metastases. *Ophthalmology*. 2016; 123: 1118–1128. 26923342 10.1016/j.ophtha.2016.01.023

[7] Ewens KG, Kanetsky PA, Richards-Yutz J, et al. Chromosome 3 status combined with BAP1 and EIF1AX mutation profiles are associated with metastasis in uveal melanoma. *Invest Ophthalmol Vis Sci*. 2014; 55: 5160–5167. 24970262 10.1167/iovs.14-14550

[8] Lamas NJ, Martel A, Nahon-Esteve S, et al. Prognostic biomarkers in uveal melanoma: the status quo, recent advances and future directions. *Cancers (Basel)*. 2021; 14: 96. 35008260 10.3390/cancers14010096 PMC8749988

[9] Cole YC, Zhang YZ, Gallo B, et al. Correlation between BAP1 localization, driver mutations, and patient survival in uveal melanoma. *Cancers (Basel)*. 2022; 14: 4105. 36077643 10.3390/cancers14174105 PMC9454448

[10] Martin M, Masshofer L, Temming P, et al. Exome sequencing identifies recurrent somatic mutations in EIF1AX and SF3B1 in uveal melanoma with disomy 3. *Nat Genet*. 2013; 45: 933–936. 23793026 10.1038/ng.2674 PMC4307600

[11] Furney SJ, Pedersen M, Gentien D, et al. SF3B1 mutations are associated with alternative splicing in uveal melanoma. *Cancer Discov*. 2013; 3: 1122–1129. 23861464 10.1158/2159-8290.CD-13-0330 PMC5321577

[12] Harbour JW, Roberson ED, Anbunathan H, Onken MD, Worley LA, Bowcock AM. Recurrent mutations at codon 625 of the splicing factor SF3B1 in uveal melanoma. *Nat Genet*. 2013; 45: 133–135. 23313955 10.1038/ng.2523 PMC3789378

[13] Alsafadi S, Houy A, Battistella A, et al. Cancer-associated SF3B1 mutations affect alternative splicing by promoting alternative branchpoint usage. *Nat Commun*. 2016; 7: 10615. 26842708 10.1038/ncomms10615 PMC4743009

[14] Nguyen JQN, Drabarek W, Yavuzyigitoglu S, et al. Spliceosome mutations in uveal melanoma. *Int J Mol Sci*. 2020; 21: 9546. 33333932 10.3390/ijms21249546 PMC7765440

[15] Szalai E, Jiang Y, van Poppelen NM, et al. Association of uveal melanoma metastatic rate with stochastic mutation rate and type of mutation. *JAMA Ophthalmol*. 2018; 136: 1115–1120. 30073324 10.1001/jamaophthalmol.2018.2986 PMC6233840

[16] Zabor EC, Radivoyevitch T, Singh AD, et al. Conditional survival in uveal melanoma. *Ophthalmol Retina*. 2021; 5: 536–542. 32979556 10.1016/j.oret.2020.09.015

[17] Drabarek W, van Riet J, Nguyen JQN, et al. Identification of early-onset metastasis in SF3B1 mutated uveal melanoma. *Cancers (Basel)*. 2022; 14: 846. 35159112 10.3390/cancers14030846 PMC8834136

[18] Bigot J, Lalanne AI, Lucibello F, et al. Splicing patterns in SF3B1-mutated uveal melanoma generate shared immunogenic tumor-specific neoepitopes. *Cancer Discov*. 2021; 11: 1938–1951. 33811047 10.1158/2159-8290.CD-20-0555

[19] Yavuzyigitoglu S, Drabarek W, Smit KN, et al. Correlation of gene mutation status with copy number profile in uveal melanoma. *Ophthalmology*. 2017; 124: 573–575. 27916271 10.1016/j.ophtha.2016.10.039

[20] Darman RB, Seiler M, Agrawal AA, et al. Cancer-associated SF3B1 hotspot mutations induce cryptic 3' splice site selection through use of a different branch point. *Cell Rep*. 2015; 13: 1033–1045. 26565915 10.1016/j.celrep.2015.09.053

[21] Lee SC, Abdel-Wahab O. Therapeutic targeting of splicing in cancer. *Nat Med*. 2016; 22: 976–986. 27603132 10.1038/nm.4165 PMC5644489

[22] Lee SC, Dvinge H, Kim E, et al. Modulation of splicing catalysis for therapeutic targeting of leukemia with mutations in genes encoding spliceosomal proteins. *Nat Med*. 2016; 22: 672–678. 27135740 10.1038/nm.4097 PMC4899191

[23] Obeng EA, Chappell RJ, Seiler M, et al. Physiologic expression of Sf3b1(K700E) causes impaired erythropoiesis, aberrant splicing, and sensitivity to therapeutic spliceosome modulation. *Cancer Cell*. 2016; 30: 404–417. 27622333 10.1016/j.ccell.2016.08.006 PMC5023069

[24] Eskens FA, Ramos FJ, Burger H, et al. Phase I pharmacokinetic and pharmacodynamic study of the first-in-class spliceosome inhibitor E7107 in patients with advanced solid tumors. *Clin Cancer Res*. 2013; 19: 6296–6304. 23983259 10.1158/1078-0432.CCR-13-0485

[25] Hong DS, Kurzrock R, Naing A, et al. A phase I, open-label, single-arm, dose-escalation study of E7107, a precursor messenger ribonucleic acid (pre-mRNA) splicesome inhibitor administered intravenously on days 1 and 8 every 21 days to patients with solid tumors. *Invest New Drugs*. 2014; 32: 436–444. 24258465 10.1007/s10637-013-0046-5

[26] Kotake Y, Sagane K, Owa T, et al. Splicing factor SF3b as a target of the antitumor natural product pladienolide. *Nat Chem Biol*. 2007; 3: 570–575. 17643112 10.1038/nchembio.2007.16

[27] Folco EG, Coil KE, Reed R. The anti-tumor drug E7107 reveals an essential role for SF3b in remodeling U2 snRNP to expose the branch point-binding region. *Genes Dev*. 2011; 25: 440–444. 21363962 10.1101/gad.2009411 PMC3049285

[28] Corrionero A, Minana B, Valcarcel J. Reduced fidelity of branch point recognition and alternative splicing induced by the anti-tumor drug spliceostatin A. *Genes Dev*. 2011; 25: 445–459. 21363963 10.1101/gad.2014311 PMC3049286

[29] Nathan P, Hassel JC, Rutkowski P, et al. Overall survival benefit with tebentafusp in metastatic uveal melanoma. *N Engl J Med*. 2021; 385: 1196–1206. 34551229 10.1056/NEJMoa2103485

[30] Amirouchene-Angelozzi N, Nemati F, Gentien D, et al. Establishment of novel cell lines recapitulating the genetic landscape of uveal melanoma and preclinical validation of mTOR as a therapeutic target. *Mol Oncol*. 2014; 8: 1508–1520. 24994677 10.1016/j.molonc.2014.06.004 PMC5528590

[31] Koopmans AE, Verdijk RM, Brouwer RW, et al. Clinical significance of immunohistochemistry for detection of BAP1 mutations in uveal melanoma. *Mod Pathol*. 2014; 27: 1321–1330. 24633195 10.1038/modpathol.2014.43

[32] Koopmans AE, Vaarwater J, Paridaens D, et al. Patient survival in uveal melanoma is not affected by oncogenic mutations in GNAQ and GNA11. *Br J Cancer*. 2013; 109: 493–496. 23778528 10.1038/bjc.2013.299 PMC3721402

[33] Felix Krueger. TrimGalore. Taking appropriate QC measures for RRBS-type or other -Seq applications with Trim Galore! User's guide. 2021, Available at: https://github.com/FelixKrueger/TrimGalore/blob/master/Docs/Trim_Galore_User_Guide.md.

[34] Gallardo C, Delisle L. Genome-wide alternative splicing analysis (galaxy training materials). Galaxy Training! 2023. Available at: https://training.galaxyproject.org/training-material/topics/transcriptomics/tutorials/differential-isoform-expression/tutorial.html#:∼:text=In%20this%20tutorial,%20we%20aim%20to%20perform%20a%20genome-wide%20analysis#:∼:text=In%20this%20tutorial,%20we%20aim%20to%20perform%20a%20genome-wide%20analysis.

[35] Hiltemann S, Rasche H, Gladman S, et al. Galaxy training: a powerful framework for teaching! *PLoS Comput Biol*. 2023; 19: e1010752. 36622853 10.1371/journal.pcbi.1010752 PMC9829167

[36] Batut B, Hiltemann S, Bagnacani A, et al. Community-driven data analysis training for biology. *Cell Syst*. 2018; 6: 752–758.e751. 29953864 10.1016/j.cels.2018.05.012 PMC6296361

[37] Mertes C, Scheller IF, Yepez VA, et al. Detection of aberrant splicing events in RNA-seq data using FRASER. *Nat Commun*. 2021; 12: 529. 33483494 10.1038/s41467-020-20573-7 PMC7822922

[38] Kollerup Madsen B, Hilscher M, Zetner D, Rosenberg J. Adverse reactions of dimethyl sulfoxide in humans: a systematic review. *F1000Res*. 2018; 7: 1746. 31489176 10.12688/f1000research.16642.1 PMC6707402

[39] Mooy CM, Luyten GP, de Jong PT, et al. Immunohistochemical and prognostic analysis of apoptosis and proliferation in uveal melanoma. *Am J Pathol*. 1995; 147: 1097–1104. 7573354 PMC1871011

[40] Nakajima H, Hori Y, Terano H, et al. New antitumor substances, FR901463, FR901464 and FR901465. II. Activities against experimental tumors in mice and mechanism of action. *J Antibiot (Tokyo)*. 1996; 49: 1204–1211. 9031665 10.7164/antibiotics.49.1204

[41] Lagisetti C, Yermolina MV, Sharma LK, Palacios G, Prigaro BJ, Webb TR. Pre-mRNA splicing-modulatory pharmacophores: the total synthesis of herboxidiene, a pladienolide-herboxidiene hybrid analog and related derivatives. *ACS Chem Biol*. 2014; 9: 643–648. 24377313 10.1021/cb400695j PMC3962696

[42] Fan L, Lagisetti C, Edwards CC, Webb TR, Potter PM. Sudemycins, novel small molecule analogues of FR901464, induce alternative gene splicing. *ACS Chem Biol*. 2011; 6: 582–589. 21344922 10.1021/cb100356k PMC3113647

[43] Shirai CL, White BS, Tripathi M, et al. Mutant U2AF1-expressing cells are sensitive to pharmacological modulation of the spliceosome. *Nat Commun*. 2017; 8: 14060. 28067246 10.1038/ncomms14060 PMC5227701

[44] Maguire SL, Leonidou A, Wai P, et al. SF3B1 mutations constitute a novel therapeutic target in breast cancer. *J Pathol*. 2015; 235: 571–580. 25424858 10.1002/path.4483 PMC4643177

[45] van Poppelen NM, Drabarek W, Smit KN, et al. SRSF2 mutations in uveal melanoma: a preference for in-frame deletions? *Cancers (Basel)*. 2019; 11: 1200. 31426461 10.3390/cancers11081200 PMC6721539

[46] Yoshida K, Sanada M, Shiraishi Y, et al. Frequent pathway mutations of splicing machinery in myelodysplasia. *Nature*. 2011; 478: 64–69. 21909114 10.1038/nature10496

[47] Yokoi A, Kotake Y, Takahashi K, et al. Biological validation that SF3b is a target of the antitumor macrolide pladienolide. *FEBS J*. 2011; 278: 4870–4880. 21981285 10.1111/j.1742-4658.2011.08387.x

[48] Tate JG, Bamford S, Jubb HC, et al. COSMIC: the catalogue of somatic mutations in cancer. *Nucleic Acids Res*. 2019; 47: D941–D947. 30371878 10.1093/nar/gky1015 PMC6323903

[49] Nemati F, de Koning L, Gentien D, et al. Patient derived xenografts (PDX) models as an avatar to assess personalized therapy options in uveal melanoma: a feasibility study. *Curr Oncol*. 2023; 30: 9090–9103. 37887557 10.3390/curroncol30100657 PMC10604955

[50] Sciarrillo R, Wojtuszkiewicz A, El Hassouni B, et al. Splicing modulation as novel therapeutic strategy against diffuse malignant peritoneal mesothelioma. *EBioMedicine*. 2019; 39: 215–225. 30581150 10.1016/j.ebiom.2018.12.025 PMC6355829

[51] Singh AD. Uveal melanoma: implications of tumor doubling time. *Ophthalmology*. 2001; 108: 829–831. 11319992 10.1016/s0161-6420(00)00607-2

[52] Eskelin S, Pyrhonen S, Summanen P, Hahka-Kemppinen M, Kivela T. Tumor doubling times in metastatic malignant melanoma of the uvea: tumor progression before and after treatment. *Ophthalmology*. 2000; 107: 1443–1449. 10919885 10.1016/s0161-6420(00)00182-2

[53] Skipper HE, Perry S. Kinetics of normal and leukemic leukocyte populations and relevance to chemotherapy. *Cancer Res*. 1970; 30: 1883–1897. 4917694

[54] Bayat M, Ghaidari D. Chapter 5 - Nanoparticles and liver cancer. In: Yadav AK, Gupta U, Sharma R (eds), *Nano Drug Delivery Strategies for the Treatment of Cancers*. New York, NY: Academic Press; 2021: 119–143.

[55] Yao T, Zhang Z, Li Q, et al. Long-read sequencing reveals alternative splicing-driven, shared immunogenic neoepitopes regardless of SF3B1 status in uveal melanoma. *Cancer Immunol Res*. 2023; 11: 1671–1687. 37756564 10.1158/2326-6066.CIR-23-0083

[56] Steensma DP, Wermke M, Klimek VM, et al. Phase I first-in-human dose escalation study of the oral SF3B1 modulator H3B-8800 in myeloid neoplasms. *Leukemia*. 2021; 35: 3542–3550. 34172893 10.1038/s41375-021-01328-9 PMC8632688

[57] Seiler M, Yoshimi A, Darman R, et al. H3B-8800, an orally available small-molecule splicing modulator, induces lethality in spliceosome-mutant cancers. *Nat Med*. 2018; 24: 497–504. 29457796 10.1038/nm.4493 PMC6730556

[58] Lopez-Oreja I, Gohr A, Playa-Albinyana H, et al. SF3B1 mutation-mediated sensitization to H3B-8800 splicing inhibitor in chronic lymphocytic leukemia. *Life Sci Alliance*. 2023; 6: e202301955. 37562845 10.26508/lsa.202301955 PMC10415613

[59] North K, Benbarche S, Liu B, et al. Synthetic introns enable splicing factor mutation-dependent targeting of cancer cells. *Nat Biotechnol*. 2022; 40: 1103–1113. 35241838 10.1038/s41587-022-01224-2 PMC9288984

[60] Ten Hacken E, Valentin R, Regis FFD, et al. Splicing modulation sensitizes chronic lymphocytic leukemia cells to venetoclax by remodeling mitochondrial apoptotic dependencies. *JCI Insight*. 2018; 3: e121438. 30282833 10.1172/jci.insight.121438 PMC6237462

[61] Mukherjee N, Dart CR, Amato CM, et al. Expression differences in BCL2 family members between uveal and cutaneous melanomas account for varying sensitivity to BH3 mimetics. *J Invest Dermatol*. 2022; 142: 1912–1922.e1917. 34942200 10.1016/j.jid.2021.11.035 PMC9635014

[62] Lappin KM, Barros EM, Jhujh SS, et al. Cancer-associated SF3B1 mutations confer a BRCA-like cellular phenotype and synthetic lethality to PARP inhibitors. *Cancer Res*. 2022; 82: 819–830. 35027467 10.1158/0008-5472.CAN-21-1843 PMC7612475

[63] de Koning L, Decaudin D, El Botty R, et al. PARP inhibition increases the response to chemotherapy in uveal melanoma. *Cancers (Basel)*. 2019; 11: 751. 31146482 10.3390/cancers11060751 PMC6628115

[64] Bland P, Saville H, Wai PT, et al. SF3B1 hotspot mutations confer sensitivity to PARP inhibition by eliciting a defective replication stress response. *Nat Genet*. 2023; 55: 1311–1323. 37524790 10.1038/s41588-023-01460-5 PMC10412459

[65] Vivet-Noguer R, Tarin M, Canbezdi C, et al. Glycolysis dependency as a hallmark of SF3B1-mutated cells. *Cancers (Basel)*. 2022; 14: 2113. 35565242 10.3390/cancers14092113 PMC9101609

[66] Porporato PE, Filigheddu N, Pedro JMB, Kroemer G, Galluzzi L. Mitochondrial metabolism and cancer. *Cell Res*. 2018; 28: 265–280. 29219147 10.1038/cr.2017.155 PMC5835768

[67] Gaude E, Frezza C. Defects in mitochondrial metabolism and cancer. *Cancer Metab*. 2014; 2: 10. 25057353 10.1186/2049-3002-2-10 PMC4108232

