# Supplementary material for: The Impact of Spliceosome Inhibition in SF3B1-Mutated Uveal Melanoma
Source: Invest Ophthalmol Vis Sci. 2024 Oct 7;65(12):11. doi: 10.1167/iovs.65.12.11 (PMC11463709; doi:10.1167/iovs.65.12.11)
Supplement: Supplement 1 [file iovs-65-12-11_s001.pdf]

Supplementary material

a

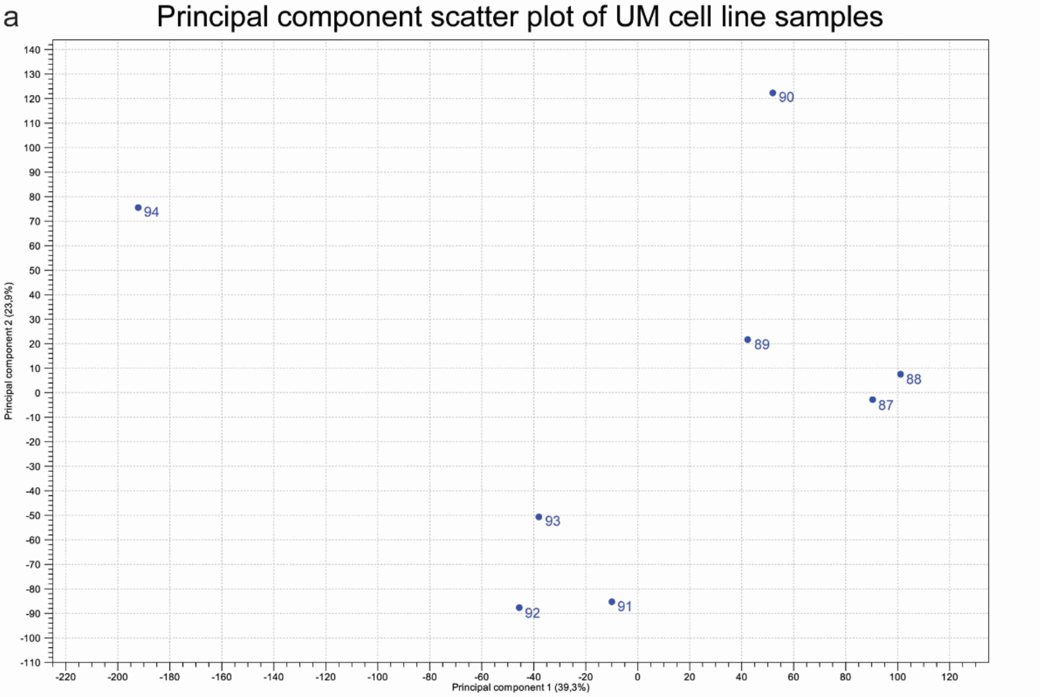
Principal component scatter plot of UM cell line samples
Principal component 2 (23,9%)
Principal component 1 (39,3%)
90
94
89
88
87
93
92
91

b

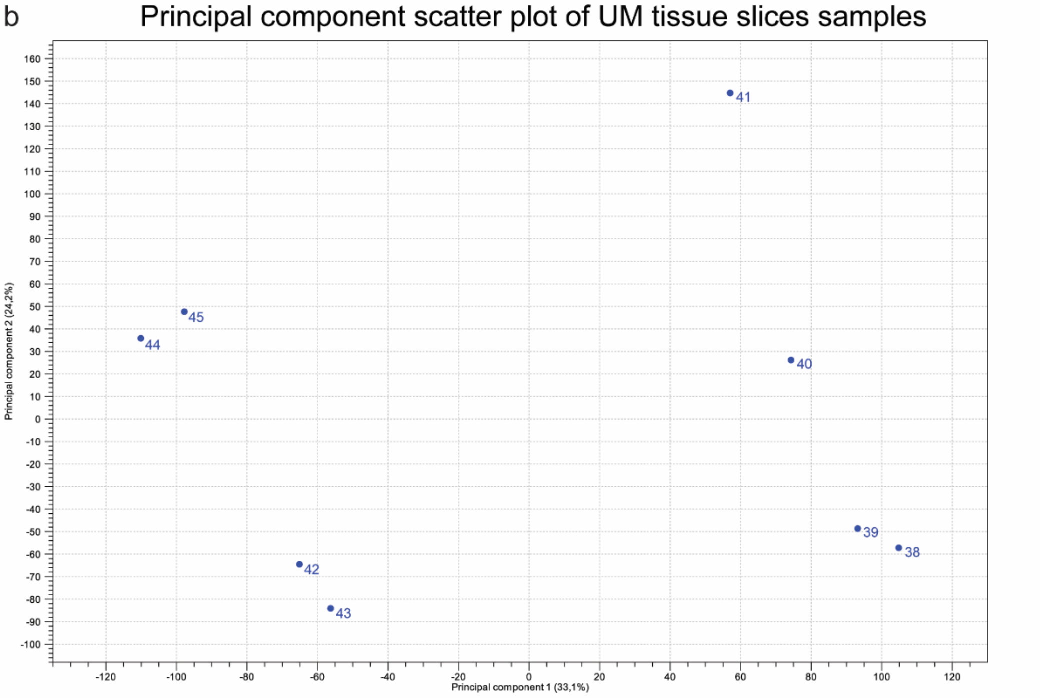
Principal component scatter plot of UM tissue slices samples
Principal component 2 (24,2%)
Principal component 1 (33,1%)
41
45
44
40
39
38
42
43

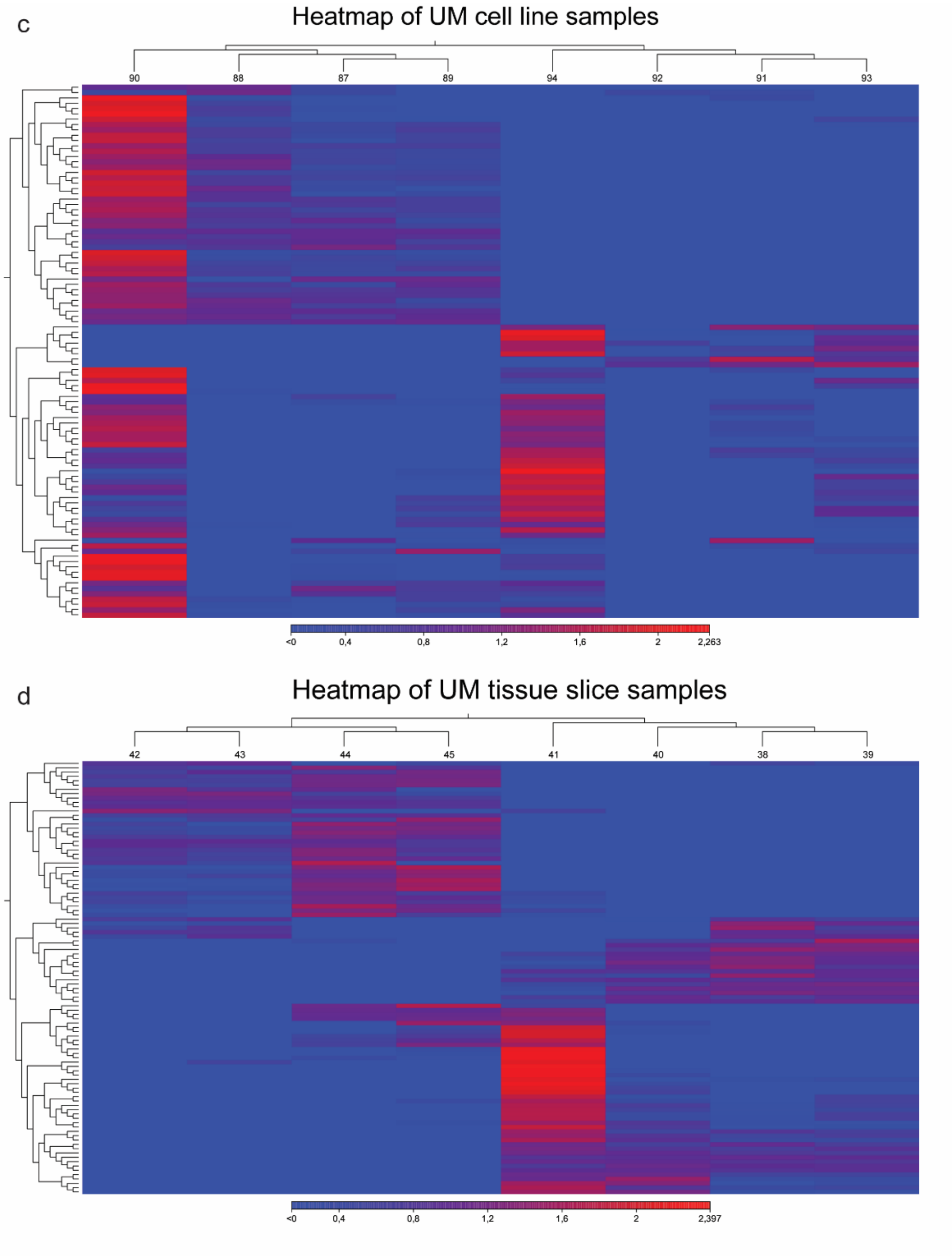

Figure S1. PCA plots and heatmaps of UM RNA-seq samples. PCA plots of (a) *SF3B1*^{MUT} and *SF3B1*^{WT} cell line samples and (b) *SF3B1*^{MUT} and *SF3B1*^{WT} tissue slice samples. Heatmaps of (c) *SF3B1*^{MUT} and *SF3B1*^{WT} cell line samples and (d) *SF3B1*^{MUT} and *SF3B1*^{WT} tissue slice samples. Samples 38-41 = *SF3B1*^{MUT} tumor slices; samples 42-45 = *SF3B1*^{WT} tumor slices; samples 87-90 = *SF3B1*^{MUT} cell line; samples 91-94 *SF3B1*^{WT} cell line (conditions see Table S2).

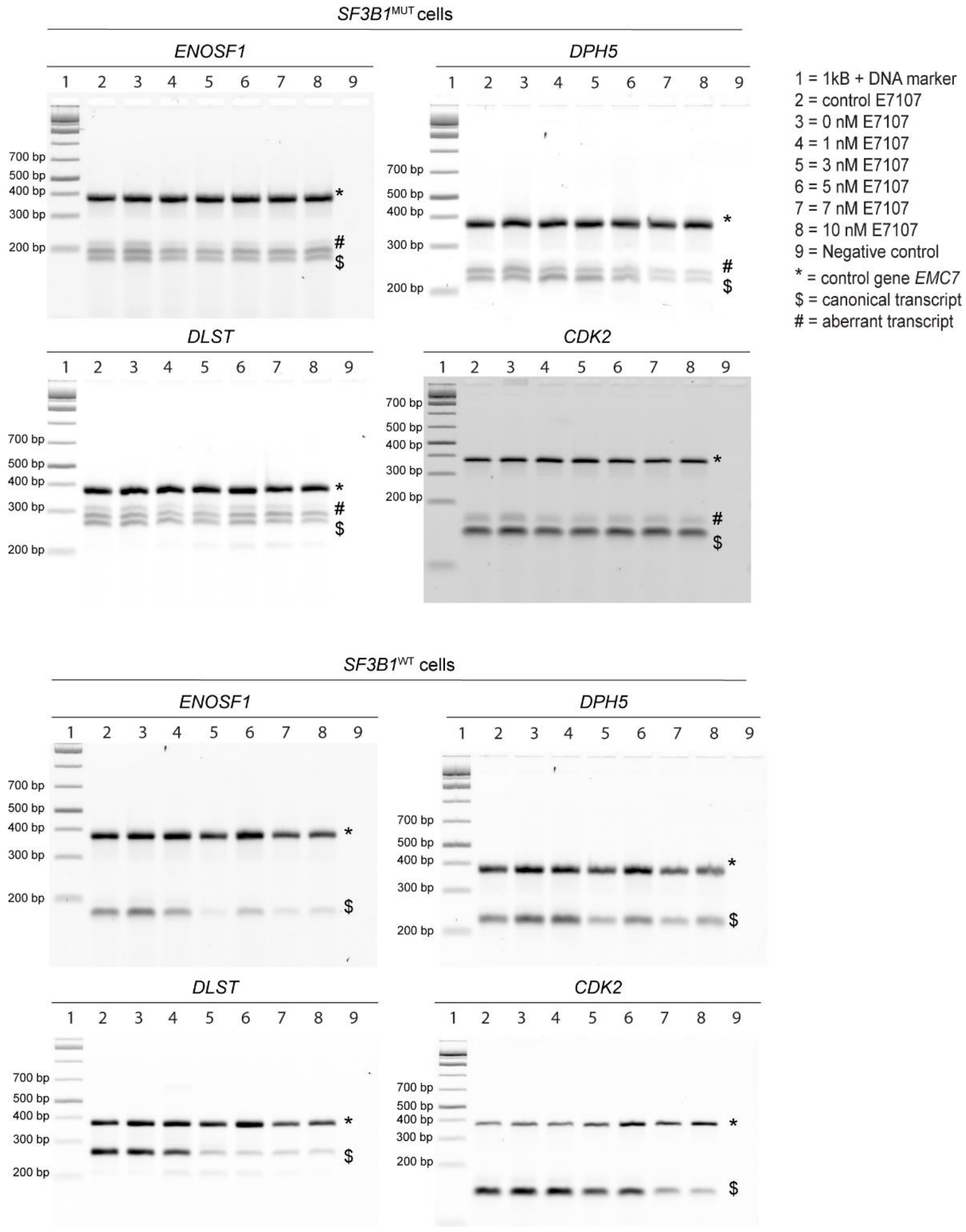

Figure S2. RT–PCR analysis of the effect of E7107 treatment on splicing inhibition in UM cell lines. Multiplex RT–PCR products for the transcription factors *ENOSF1*, *DPH5*, *DLST* and *CDK2* in Mel202 *SF3B1*^{MUT} and 92.1 *SF3B1*^{WT} cell lines treated with different concentrations of E7107. Amplification of the *SF3B1*^{MUT}-sensitive genes resulted in both aberrant and canonical transcript formation for Mel202 *SF3B1*^{MUT} cells, whereas the 92.1 *SF3B1*^{WT} products only displayed canonical transcript formation. The insensitive gene *EMC7* was used as a loading control for both cDNA quality and quantity.

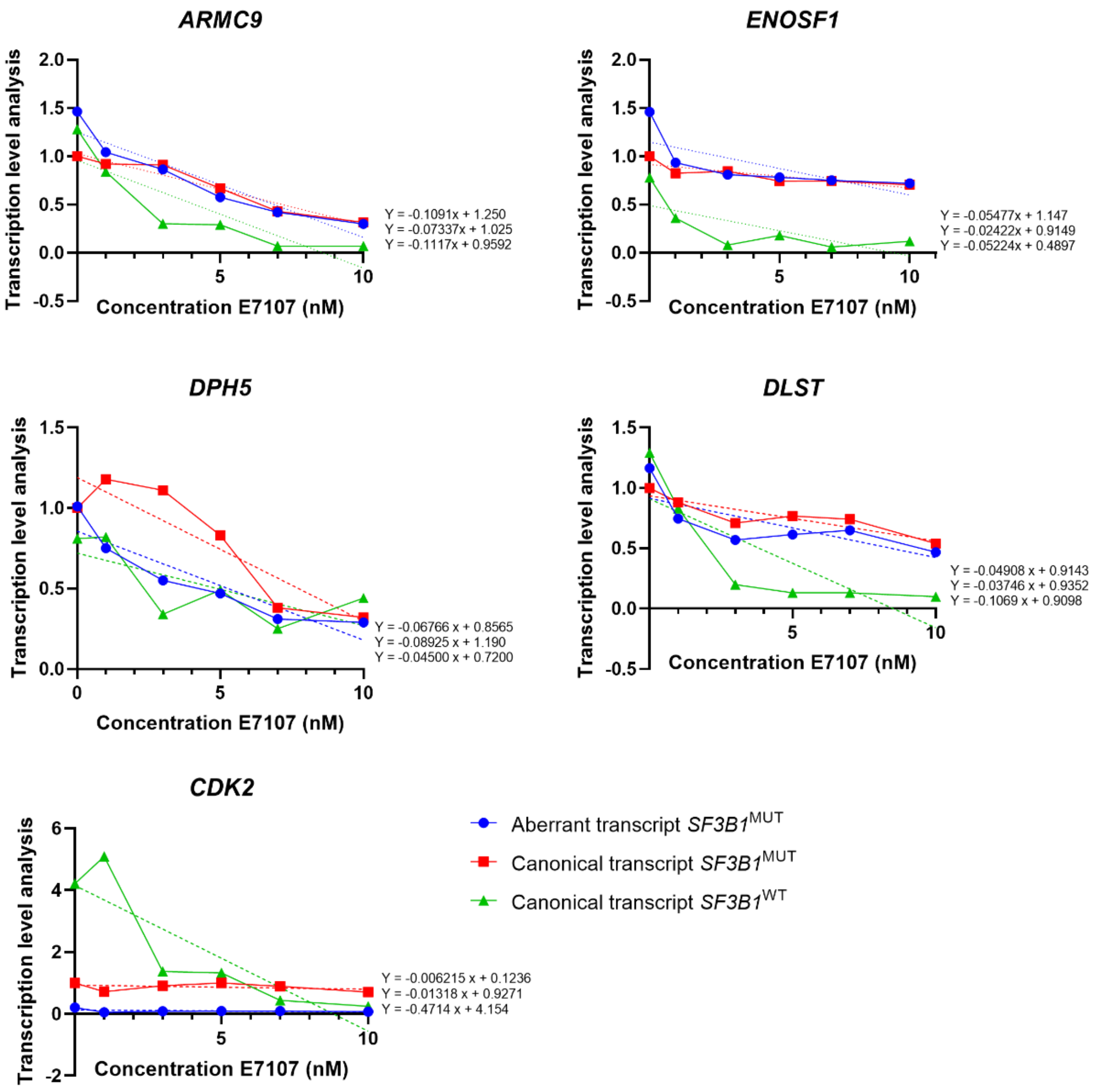

Figure S3. Quantification of the preferential sensitivity of Mel202 *SF3B1*^{MUT} and 92.1 *SF3B1*^{WT} UM cell lines to E7107. Aberrant and canonical transcript formation was quantified using ImageQuantTL. Transcription level analysis of *ARMC9*, *ENOSF1*, *DPH5*, *DLST* and *CDK2* in UM cell lines Mel202 and 92.1. Linear regression analysis was used to determine the degree of splicing inhibition (regression comparison between aberrant and canonical transcripts in Mel202 *SF3B1*^{MUT} UM cells: *ARMC9*, p = 0.1195; *ENOSF1*, p = 0.2730; *DPH5*, p = 0.4093; *DLST*, p = 0.6249; *CDK2*, p = 0.6989).

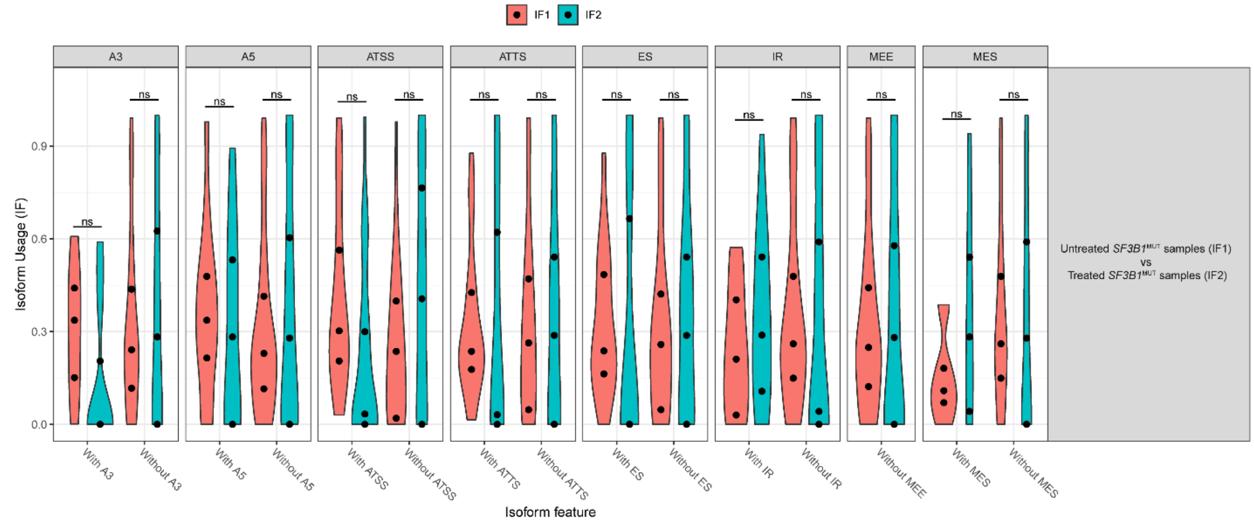

Figure S4. Genome-wide changes in isoform usage in a violin plot. No significant difference in isoform usage was found genome-wide between untreated and treated *SF3B1*^{MUT} samples. The dots in the violin plots indicate the 25th, 50th (median), and 75th percentiles. A3; alternative 3' splice site, A5; alternative 5' splice site, ATSS; alternative transcription start site, ATTS; alternative transcription termination site, ES; exon skipping, IR; intron retention, MEE; mutually exclusive exons, MES; multiple exon skipping, IS; isoform usage, ns; not significant.

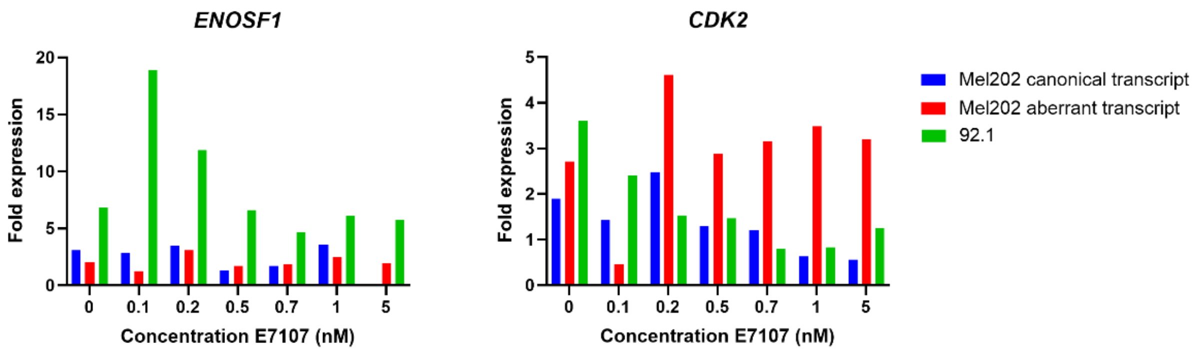

Figure S5. Quantitative PCR of *ENOSF1* and *CDK2* in *SF3B1*^{MUT} and *SF3B1*^{WT} UM cells treated with E7107. The relative fold changes in the expression levels of canonical and aberrant transcripts of *ENOSF1* and *CDK2* in *SF3B1*^{MUT} and *SF3B1*^{WT} cells were determined. The results are the average of three replicates. No differences were found between the cell lines.

Table S1. Primer sequences.

Gene	Forward	Reverse
ARMC9	GACATCATGGAAGCCGATCTG	CCTTGTCTTCCCCGTGTTG
ENOSF1	GATGACATGCGAAGATGCCA	GCTTGAACTTGGCCAGCTT
DPH5	TGGACAGACACTTGGAGACC	GGGCTGCTTGGTTTACACTC
DLST	AGTGTCTTCAGTGTTCGCTT	GCTTCAATCACGCCATTTGC
CDK2	GGATGCCTCTGCTCTCACTG	AATGGCAGAAAGCTAGGCCC

Table S2. RNA sequencing alignment metrics. CL = cell line; TS = tumor slice.

Sample	Condition	Source	Read counts	Paired, mapped pairs (%)	Paired, broken pairs (%)	Paired, not mapped (%)	Mapped to genes (%)
**38**	*SF3B1*^{MUT} Medium TS	Tumor 014	149,922,012	93.99	3.39	2.63	97.57
**39**	*SF3B1*^{MUT} 0 nM E7107 TS	Tumor 014	81,519,640	94.68	3.07	2.25	97.88
**40**	*SF3B1*^{MUT} 1 nM E7107 TS	Tumor 014	101,396,164	94.31	3.44	2.25	98.04
**41**	*SF3B1*^{MUT} 5 nM E7107 TS	Tumor 014	87,217,358	93.24	3.78	2.99	97.35
**42**	*SF3B1*^{WT} Medium TS	Tumor 006	107,258,286	93.58	4.34	2.08	98.25
**43**	*SF3B1*^{WT} 0 nM E7107 TS	Tumor 006	90,112,262	95.04	2.95	2.01	98.03
**44**	*SF3B1*^{WT} 1 nM E7107 TS	Tumor 006	101,469,750	94.03	3.75	2.22	98.29
**45**	*SF3B1*^{WT} 5 nM E7107 TS	Tumor 006	90,129,936	93.73	3.61	2.66	97.54
**87**	*SF3B1*^{MUT} Medium CL	Mel202	71,577,654	95.98	1.60	2.42	98.55
**88**	*SF3B1*^{MUT} 0 nM E7107 CL	Mel202	87,036,100	95.46	2.38	2.16	98.75
**89**	*SF3B1*^{MUT} 1 nM E7107 CL	Mel202	72,292,188	95.63	1.84	2.53	98.52
**90**	*SF3B1*^{MUT} 5 nM E7107 CL	Mel202	169,070,054	94.74	3.04	2.22	98.80
**91**	*SF3B1*^{WT} Medium CL	92.1	67,958,438	95.60	2.02	2.37	98.74
**92**	*SF3B1*^{WT} 0 nM E7107 CL	92.1	88,471,060	95.65	2.18	2.17	98.91
**93**	*SF3B1*^{WT} 1 nM E7107 CL	92.1	99,274,780	95.64	2.29	2.07	98.96
**94**	*SF3B1*^{WT} 5 nM E7107 CL	92.1	79,667,126	94.11	3.12	2.78	98.12

Table S3. Immunohistochemistry (IHC) settings. AB = antibody.

Antibody	Type	Company	Clone	Procedure	AB incubation time
**Melan-A**	Anti-mouse	Ventana	A103	Ultraview CC1 36’	28 minutes
**BAP1**	Anti-mouse	Santa Cruz	C-4	Ultraview CC1 64’	32 minutes
**MIB-1**	Anti-rabbit	Ventana	30-9	Ultraview CC1 32’	32 minutes
**Caspase-3**	Anti-rabbit	Ventana	5A1E	Ultraview CC1 64’	32 minutes

Table S4. UM, tumor mutational status. * = wild-type for *GNAQ* and *GNA11*; † = wild-type for *SF3B1*, *EIF1AX* or BAP1 and BAP1 IHC positive.

Tumor	Primary driver mutation	Secondary driver mutation	Tumor location
01	*GNAQ* (c.626A>T)	*SF3B1* (c.1874G>A; R625L)	choroid
02	*GNA11* (c.626A>T)	BAP1 IHC neg	choroid
03	*GNA11* (c.626A>T)	*EIF1AX* (c.25G>C)	choroid
04	*GNAQ* (c.626A>C)	*EIF1AX* (c.28A>G)	choroid
05	*GNAQ* (c.626A>T)	†	choroid
06	*GNA11* (c.626A>T)	*BAP1* (c.1985_1988del) and BAP1 IHC neg	choroid
07	*GNA11* (c.626A>T)	*BAP1* (c.1754_1755insT) and BAP1 IHC neg	choroid
08	*GNAQ* (c.626A>C)	*EIF1AX* (c.5C>T)	choroid
09	*GNAQ* (c.626A>C)	BAP1 IHC neg	ciliary body

10	*GNA11* (c.626A>T)	*BAP1* (c.68-2A>G) and BAP1 IHC neg	choroid
11	*PLCB4* (c.1888_1889delinsTT)	*SF3B1* (c.1873C>T; R625C)	choroid
12	*	*BAP1* (c.1878del) and BAP1 IHC neg	choroid
13	*GNAQ* (c.626A>T)	*BAP1* (c.1379C>G) and BAP1 IHC neg	choroid
14	*GNAQ* (c.626A>C)	*SF3B1* (c.1873C>T; R625C)	choroid
15	*GNA11* (c.626A>T)	*EIF1AX* (c.17-2A>C)	choroid
16	*GNA11* (c.626A>T)	*EIF1AX* (c.11A>G)	choroid
17	*GNAQ* (c.626A>T)	*BAP1* (c.1951-1955delinsG) and BAP1 IHC neg	choroid
18	*GNA11* (c.626A>T)	†	choroid
19	*GNA11* (c.626A>T)	*BAP1* (c.550G>T) and BAP1 IHC neg	ciliary body
20	*CYSLTR2* (c.386T>A)	*BAP1* (c.691_705del) and BAP1 IHC neg	ciliary body

Table S5. Top 10 genes with isoform switching with predicted functional consequences.

Genes
AGAP4
TUBA1B
EIF3C
TUBA1C
PMS2
TUBA1A
SNHG16
GTF2IP1
IKBKG
H2BC5
